# An Integrated Electrolysis-Enrichment Microchip for Ultra-Rapid and Sensitive mRNA Detection

**DOI:** 10.34133/research.0928

**Published:** 2025-10-09

**Authors:** Long Cheng, Zhiying Wang, Chengbao Wu, Feng Liu, Hui Li, Yunke Feng, Xi Chen, Xinxin Hang, Yu Zeng, Wei Mu, Yuhao Zhou, Liye Liu, Lingqian Chang, Qiaowei Liu, Yi Hu, Yang Wang

**Affiliations:** ^1^ School of Biomedical Engineering, Anhui Medical University, Hefei 230032, China.; ^2^ Key Laboratory of Biomechanics and Mechanobiology, Ministry of Education, Beijing Advanced Innovation Center for Biomedical Engineering, Beihang University, Beijing 100191, China.; ^3^ School of Biological Science and Medical Engineering, Beihang University, Beijing 100191, China.; ^4^ Department of Oncology, the Fifth Medical Center, Chinese PLA General Hospital, Beijing 100071, China.; ^5^ Department of Ophthalmology, Beijing Friendship Hospital, Capital Medical University, Beijing 100050, China.; ^6^ School of Engineering Medicine, Beihang University, Beijing 100191, China.; ^7^ Medical School of Chinese PLA, Beijing 100853, China.

## Abstract

Messenger RNA (mRNA) detection plays a vital role in gene expression analysis, disease diagnosis, and precision medicine. However, current methods are limited by complex chemical-based extraction procedures and the rapid degradation of mRNA. To overcome these challenges, we developed an electrolysis-enrichment microfluidic chip (EEMC) that employs electricity-driven physical methods for ultra-rapid, efficient mRNA extraction directly from whole blood, eliminating the need for chemical reagents that can inhibit downstream analysis. By integrating electrolytic lysis with ion concentration polarization (ICP) enrichment, the EEMC completes the entire process within 10 min—6 times faster than conventional techniques, greatly avoiding the potential degradation. It achieves a high recovery efficiency of 90% to 95%, which surpasses the 85% to 90% efficiency of commercial RNA extraction kits. Combined with downstream loop-mediated isothermal amplification (LAMP), the EEMC provides a powerful solution for rapid mRNA processing and detection. Its efficacy was validated in radiation exposure models through the detection of key mRNA biomarkers (BAX, CDKN1A, and GADD45A). This environmentally friendly and high-performance platform advances point-of-care precision diagnostics.

## Introduction

Messenger RNA (mRNA) serves as a crucial bridge between genetic information and biological function, with its core significance lying in its ability to dynamically reflect gene expression changes [1]. Consequently, mRNA detection plays a critical role in regulating gene expression, enabling disease diagnosis and subtyping, supporting prognostic evaluation, and advancing personalized medicine [2,3]. However, mRNA is highly susceptible to rapid degradation by endogenous RNases, often within minutes of cell lysis, posing challenges for reliable detection [4]. Degradation can lead to the loss of target sequences—particularly at the 3′ end—hindering primer or probe binding in quantitative polymerase chain reaction (qPCR) or RNA-Seq [5]. Additionally, uneven degradation (e.g., longer fragments being more vulnerable) can compromise accurate quantification of transcript abundance [6].

Current mRNA extraction techniques (such as the TRIzol method) from whole blood typically involve chemical lysis followed by purification using magnetic beads or silica membranes [4,7]. These processes rely on toxic chemical reagents and involve cumbersome multi-step purification procedures, taking approximately 1.5 to 3 h in total [5]. This poses significant challenges to operational safety, environmental impact, and experimental outcomes [2]. The lengthy and complex procedures can lead to mRNA loss or degradation, resulting in the loss of low-concentration mRNA and affecting downstream precise quantitative detection [6]. Even with RNase inhibitors, high endogenous RNase activity often results in rapid degradation. Additionally, residual phenol or salt ions can inhibit the activity of reverse transcriptase or Taq polymerase, leading to amplification failure [5,8]. Currently, the application of integrated microfluidic chips can streamline experimental workflows to some extent, enhancing speed and portability [6]. However, their core processes still depend on traditional reagents and conventional methods, constraining mRNA extraction speed and efficiency while introducing risks of cross-reactivity that potentially compromise result accuracy. Further advancements are needed to develop ultra-fast, highly efficient, and fully integrated mRNA extraction and detection platforms [9].

To address limitations of chemical-based mRNA extraction, ion concentration polarization (ICP)-based methods have emerged as a promising technique for biomolecular separation. By generating ion concentration gradients under an electric field, ICP enables label-free and reagent-free enrichment of charged biomolecules, thereby avoiding chemical interference with downstream analysis [10]. For example, microfluidic platforms utilizing ICP have successfully separated intracellular components and ctDNA from simplified samples [11,12]. In addition, some microfluidic platforms have applied ICP to achieve continuous electrolysis of pathogens [13]. However, these existing ICP-based approaches typically rely on off-chip pretreatment and post-detection techniques, and thus lack full integration of lysis, enrichment, and detection. This gap underscores the need for a fully integrated, reagent-free, and ultra-fast ICP platform capable of addressing the high instability and low abundance of mRNA.

**Fig. 1.**
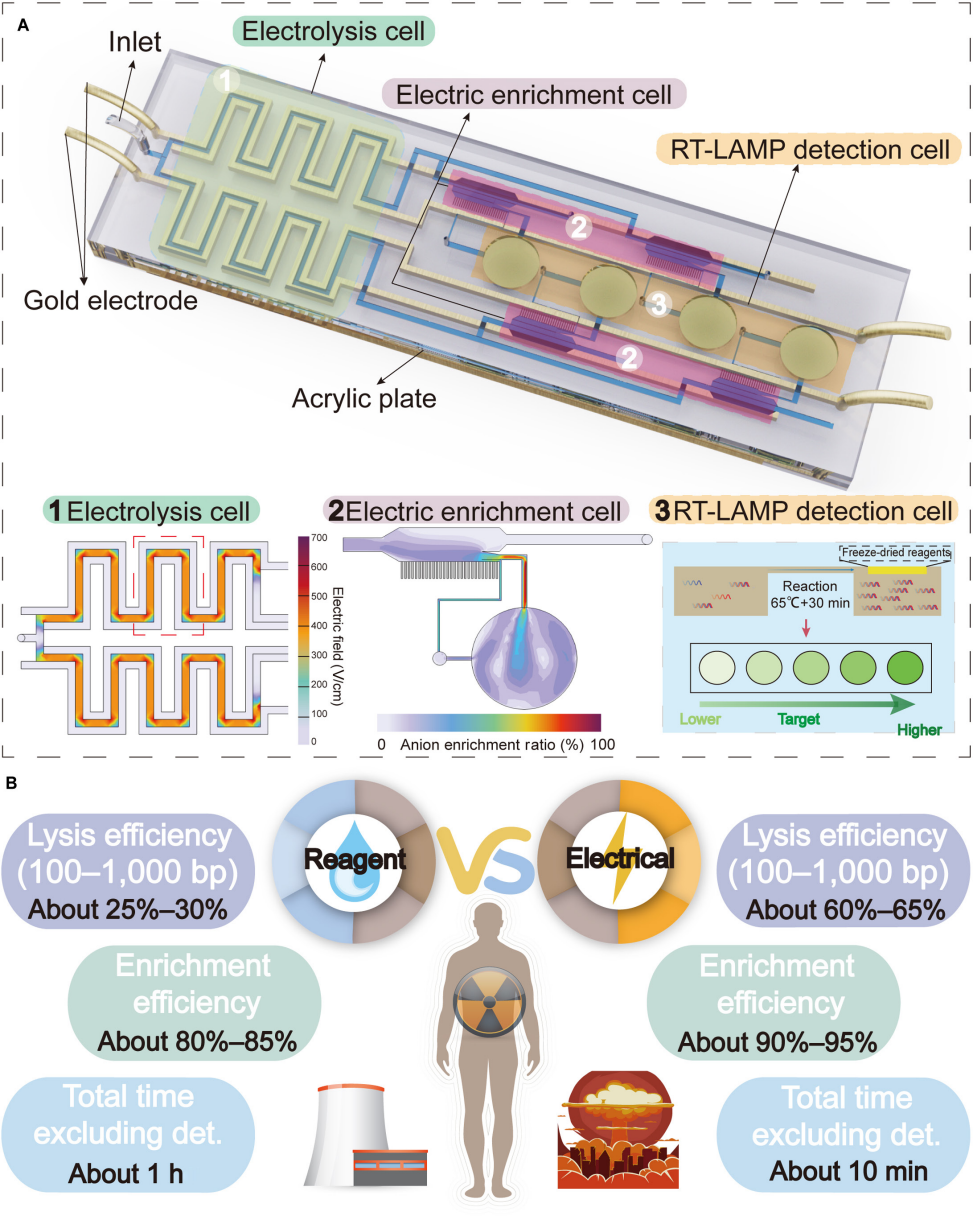
Detailed structure and functional features of the EEMC. (A) Illustration of the structure of the EEMC. The chip comprises 3 function cells: an electrolysis cell, an electric enrichment cell, and a LAMP detection cell. (1) Electric field simulation results in the electrolysis region using COMSOL Multiphysics. (2) The simulated anion mobility profile in electric enrichment cell using COMSOL Multiphysics. (3) Illustration of the LAMP detection process. (B) Comparison of the performance of reagent-based versus EEMC-based processing techniques in terms of lysis efficiency, enrichment efficiency, and total processing time.

In response to these challenges, we developed an electrolysis-enrichment microfluidic chip (EEMC) that revolutionizes mRNA processing through electricity-based physical methods [4]. The platform features an electrolytic lysis system achieving 90% to 95% lysis efficiency in just 10 min, higher than 85% to 90% of commercial RNA extraction kits, while generating optimally sized nucleic acid fragments (60% to 65% in the 100 to 1,000 bp range) and eliminating chemical inhibitors. Coupled with an ICP enrichment system, the EEMC incorporates 4 parallel processing modules with a unique “M”-shaped outlet design that enhances separation efficiency. This innovative design enables spatial separation of proteins (3× height differential) to prevent RNase degradation while delivering 10-fold concentration with <5% sample loss. The integrated EEMC platform demonstrates exceptional performance with a total processing time of 10 min (6× faster than conventional methods), 90% to 95% recovery efficiency from whole blood samples, and compatibility with various amplification techniques (qPCR and loop-mediated isothermal amplification [LAMP]), as validated in radiation exposure assessment applications, where the mRNA concentration of BAX, CDKN1A, and GADD45A in leukocytes is critical for evaluating radiation-induced damage and guiding emergency responses (Fig. 1 and Fig. S1) [8,14,15].

By replacing chemical processing with precisely controlled physical mechanisms, the EEMC platform overcomes the fundamental limitations of current mRNA extraction methods, enabling ultra-rapid processing critical for clinical decision-making, high-integrity mRNA recovery essential for accurate diagnostics, environmentally friendly operation without hazardous chemicals, and broad applicability from research to point-of-care settings [16–18]. This technological advancement represents a significant leap forward in molecular diagnostics, with particular promise for precision medicine applications requiring rapid, reliable mRNA analysis. The platform’s ability to maintain mRNA integrity while dramatically reducing processing time addresses a critical unmet need in both research and clinical environments, opening new possibilities for real-time gene expression monitoring and rapid diagnostic applications [19–21].

## Results and Discussion

### Structure and working principle of the EEMC

The EEMC’s detailed architecture and dimension are illustrated in Fig. 1A and Fig. S2, featuring 3 functionally integrated units: the electrolysis cell (green) for cell lysis, the electro-enrichment cell (purple) for mRNA concentration, and the LAMP detection cell (yellow) for final analysis. These components are fabricated by bonding 2 acrylic plates with precisely aligned microfluidic channels that enable seamless fluidic control and sample processing (Fig. S3). Two pairs of gold electrodes are positioned along the microfluidic network to provide the necessary power for both electrolysis and electro-enrichment processes. The sample processing workflow begins with whole blood introduction through the inlet, where it splits into 2 parallel streams for simultaneous electrolytic lysis in corresponding regions. The lysed samples then flow into the electro-enrichment zone for nucleic acid concentration before entering 4 parallel amplification chambers for final detection. The design enables simultaneous detection and quantification of multiple mRNA biomarkers from a single clinical sample. This capability is particularly important for comprehensive radiation damage assessment, where multiple biomarker readouts are required for accurate evaluation.

To ensure optimal device performance, we conducted COMSOL Multiphysics simulations analyzing both flow velocity distribution (Fig. S4A) and electric potential profiles (Fig. S4B), which critically influence detection performance. These simulations guided the microfluidic channel design and circuit layout optimization. The final design achieves an electric field strength of approximately 400 V/cm throughout the flow channels, with detailed analysis of the electrolysis cell’s critical region (red frame in Fig. 1A) demonstrating a stable field strength of 375 V/cm in the main channel—fully meeting the requirements for efficient cell lysis (Fig. 1A). Additional simulations of anion enrichment rates in the enrichment zone further validated the theoretical feasibility of our electro-enrichment approach (Fig. 1A). The final detection phase incorporates preloaded lyophilized LAMP reagents for isothermal amplification (65 °C for 30 min) with endpoint fluorescence detection. This integrated design combines parallel processing capability (with 2 electrolysis units, 4 electro-enrichment modules, and 4 detection chambers) with precisely controlled field strengths for each processing step, all validated through rigorous Multiphysics simulation.

### Investigation and optimization of electrolysis performance

**Fig. 2.**
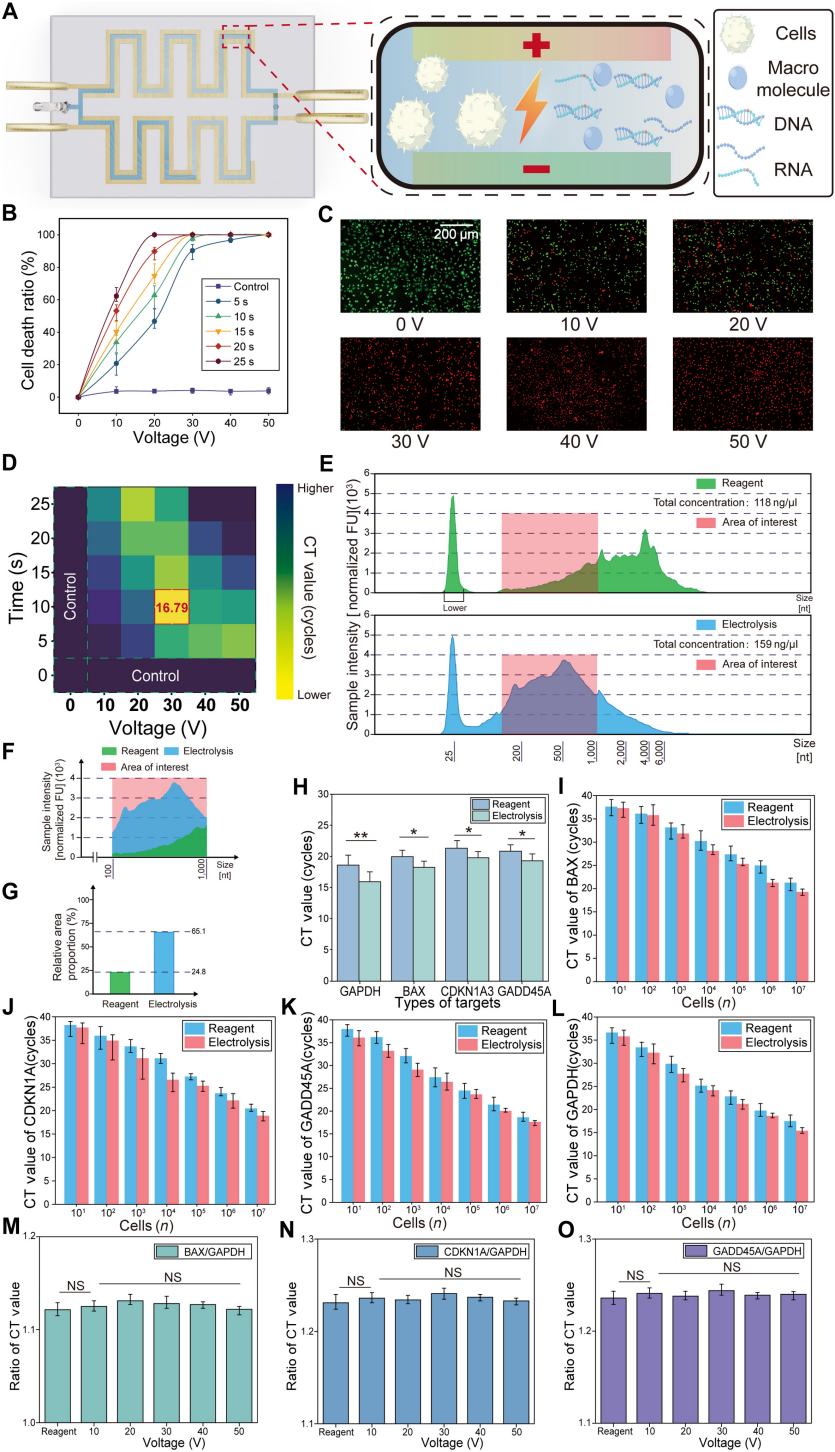
Optimization and results of electrolysis experiments. (A) Schematic of the electrolysis process: as white blood cells flow through the electrolysis zone, a high electric field induces membrane rupture, releasing intracellular macromolecules, including nucleic acids. (B) Cell death ratios observed under various voltages (0 to 50 V) and durations (0 to 25 s). (C) Fluorescence microscopy images of cells subjected to electrolysis for 10 s at different voltages (0 to 50 V). Green fluorescence (AM dye) marks live cells; red fluorescence (PI dye) marks dead cells. (D) qPCR amplification results of samples treated under different lysis conditions, showing the smallest Ct values of 16.79 at 30 V for 10 s. (E) Capillary electrophoresis comparison of nucleic acid fragments released via reagent-based lysis versus electrolysis. (F) Magnified view of capillary electrophoresis data within the 100 to 1,000 bp region of interest. (G) Quantitative comparison of the proportion of the region of interest (100 to 1,000 bp) to the total area for the 2 lysis methods. (H) Comparison of Ct values under optimal lysis conditions (30 V, 10 s) for 4 targets: GAPDH, BAX, CDKN1A, and GADD45A (*n* = 3). (I to L) Comparisons of Ct values for BAX (I), CDKN1A (J), GADD45A (K), and GAPDH (L) targets from samples processed via reagent-based and electrolysis-based lysis at varying cell concentrations ranging from 10^1^ to 10^7^; each experiment was conducted 3 times. (M to O) Ratios of BAX/GAPDH (M), CDKN1A/GAPDH (N), and GADD45A/GAPDH (O) Ct values, illustrating efficient gene-specific nucleic acid release through electrolysis (*n* = 3) (NS:P>0.05;∗:0.01<P≤0.05;∗∗:0.001<P≤0.01;∗∗∗:P≤0.001).

As whole blood flows through the microfluidic channel, white blood cell membranes rupture under a high electric field, releasing intracellular proteins and nucleic acids (Fig. 2A). Briefly, under the action of a vertical electric field, the Nafion cation-selective membrane drives negatively charged molecules to migrate toward the cathode, generating an ion enrichment zone. Meanwhile, compared with proteins or polysaccharides, nucleic acids (mRNA/DNA) have a higher charge-to-mass ratio (smaller size and linear structure), which results in faster migration and preferential enrichment in the target zone. Subsequently, an “M”-shaped outlet is configured according to the location of nucleic acid enrichment, creating a height difference between the nucleic acid channel and waste channels. Due to slower flow velocity and weaker electric field traction, larger negatively charged molecules (e.g., proteins) are retained in the waste stream, while smaller nucleic acids (100 to 1,000 bp) are effectively separated and enriched [6]. To investigate its mechanism and performance within the EEMC, we examined the effects of voltage, channel width, and flow rate that may influence the applied electric field and processing time. To assess lysis efficiency, we evaluated cell viability using fluorescence staining—Calcein-AM (494/517 nm) for live cells (green) and propidium iodide (PI) (535/617 nm) for dead cells (red). With a microfluidic channel of 0.8 mm in width, the EEMC showed a progressive increase in cell mortality with a higher voltage and a longer processing time (Fig. 2B). Due to the safety voltage constraint (35 V), we focused on 30 V and found that nearly 100% of the cells lysed within 10 s, as confirmed by fluorescence imaging (Fig. 2C). At 30 V, the electrolysis efficiency showed no significant difference between 0.8 and 0.4 mm channel width (*P* ≥ 0.05), whereas significant differences were observed compared to widths of 1.2, 1.6, and 2.0 mm (Fig. S5). For ease of fabrication, 0.8 mm was selected as the final channel width.

To validate nucleic acid release, we performed qPCR on samples processed under different conditions. At 30 V for 10 s, the cycle threshold (Ct) value reached its lowest point (16.79), indicating the highest nucleic acid extraction efficiency (Fig. 2D and Fig. S6). This may be due to the fact that excessively high voltage and prolonged processing may not only lyse cells but also degrade released nucleic acids. Accordingly, a flow rate of 120 μl/min was selected for the EEMC (Fig. S7). Under this condition, the sample passes through the microfluidic channel and remains in the lysis zone for approximately 10 s.

To further assess nucleic acid integrity, we conducted capillary electrophoresis and compared the results with those from a commercial kit (Fig. 2E). Before capillary electrophoresis detection, we used an enzyme-linked instrument to measure the purity of nucleic acids in the samples, with the results shown in Table S1. The 260/230 and 260/280 absorbance ratios indicated high purity of nucleic acids and supported the reliability of the experimental data. The red region (100 to 1,000 nt) usually represents the target range suitable for amplification and detection [6,22]. For better visualization and comparison, we segmented this region (Fig. 2F). By integrating the curves, we quantified the area of the region of interest (ROI) and the total area for reagent-based lysis and electrolysis, respectively, and calculated their ratios. The results showed that the ROI for reagent-based lysis accounted for 24.8% of the total area, while that for electrolysis accounted for 65.1% of the total area (Fig. 2G).

Based on the optimized EEMC, we first performed electrolysis, then purified the lysate with a commercial kit, and finally conducted qPCR. Compared to using a commercial kit alone for lysis and purification, the EEMC produced lower Ct values across all 4 target genes, BAX, CDKN1A, GADD45A, and GAPDH (Fig. 2H), indicating a higher efficiency of electrolysis. Additionally, we validated electrolysis efficiency across a range of white blood cell concentrations (10^1^ to 10^7^ cells). The results exhibited a linear correlation between detection metrics and cell concentration (Fig. S8). At each concentration, electrolysis demonstrated higher sensitivity than the commercial kit for detecting BAX, CDKN1A, GADD45A, and GAPDH (Fig. 2I to L).

Since the 3 target genes are associated with apoptosis and repair, we analyzed their expression ratios relative to the reference gene to assess whether electrolysis affects their expression levels (Fig. 2M to O). The results indicate that electrolysis does not directly promote or suppress the expression of these genes, supporting its superior lysis performance without altering gene expression compared to reagent-based lysis.

### Investigation of electro-enrichment performance

**Fig. 3.**
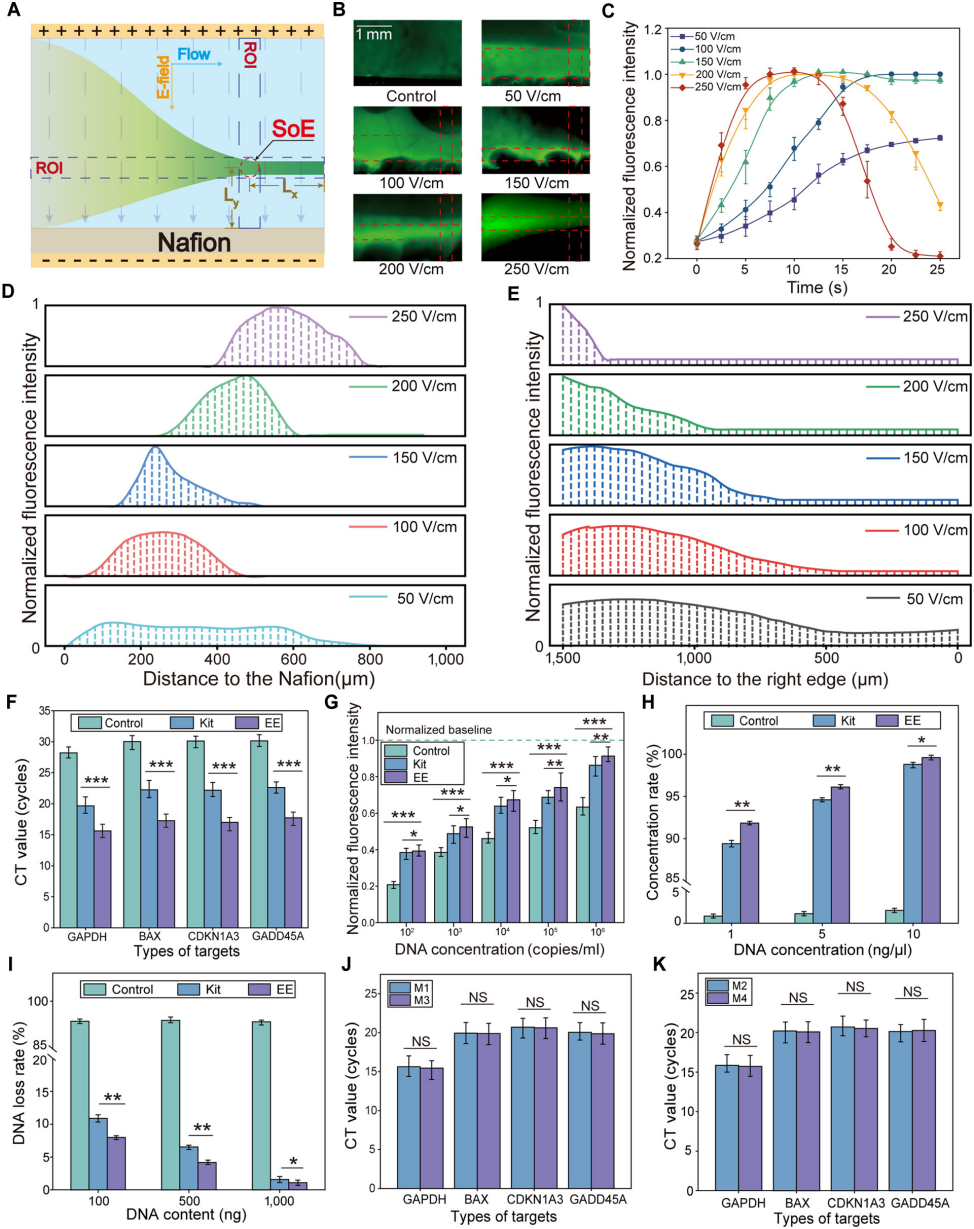
Electro-enrichment mechanism and performance analysis. (A) Schematic illustration of electro-enrichment process. Under the combination effect of a vertical electric field and cation selective transmembrane, the nucleic acids (negative charges) were enriched from the site of enrichment (SOE). The surrounding horizontal and vertical areas define the region of interest (ROI). (B) Fluorescent images of ROI under different electric field strengths ranging from 0 to 250 V/cm. (C) Enrichment efficiency as a function of electric field strength and duration. (D) Vertical fluorescence intensity profiles above the Nafion layer (Ly) under different electric field strengths from 50 to 250 V/cm. (E) Horizontal fluorescence distribution at the enrichment site (Lx) under different electric field strengths. (F) qPCR results comparing nucleic acid purification via reagent kit and electro-enrichment. (*n* = 3). (G) Fluorescence intensity at different DNA concentrations processed via reagent kit and electro-enrichment (*n* = 3). (H) Enrichment efficiency across varying DNA concentrations using reagent-based and electro-enrichment methods. Control group: sample passed through the chip without enrichment but collected at a volume equivalent to the electro-enriched group. (*n* = 3) (I) DNA loss rate across different DNA concentrations from 0.1 to 1 μg (*n* = 3). (J) Comparison of Ct results between the 2 upstream modules (M1 and M2, ordered left to right as M1, M2, M3, and M4). No significant differences observed in BAX, CDKN1A, GADD45A, and GAPDH results. (K) Comparison of Ct results between the 2 downstream modules. No significant differences observed in BAX, CDKN1A, GADD45A, and GAPDH results (NS:P>0.05;∗:0.01<P≤0.05;∗∗:0.001<P≤0.01;∗∗∗:P≤0.001).

The electro-enrichment mechanism of the EEMC builds on our previous work and operates via the dynamic ion concentration polarization (dICP) principle (Fig. 3A and Fig. S9) [23]. Here, we redesigned the enrichment zone to reduce its size while maintaining the required electric field strength essential for enrichment. This allowed for rapid electro-enrichment at a lower working voltage, significantly mitigating water electrolysis and thereby enhancing system stability (Movie S1).

To determine the optimal electric field strength, we monitored the enrichment behavior of FAM-labeled nucleic acids using fluorescence microscopy under different electric field strengths ranging from 50 to 250 V/cm as they entered the enrichment zone. The ROI highlighted the nucleic acid aggregation path, indicating the successful enrichment (Fig. 3B). Results showed that higher electric field strengths accelerated enrichment and increased the concentration ratio, while lower strengths delayed enrichment and reduced the concentration efficiency (Fig. 3C). Additionally, higher strength caused Ly (the vertical distance to the Nafion membrane) to shift upward (Fig. 3D) and Lx (the horizontal distance from the right outlet) to move leftward (Fig. 3E). This is due to the fact that a greater electric field force leads to faster ions migrating speed, therefore leading to a quicker formation of an ion concentration gradient and an earlier appearance of the enrichment point. However, excessively high electric field strength generates rapid ion migration, thereby disrupting the concentration gradient and causing the enrichment zone to become unstable, leading to premature dispersion, consistent with our previous findings (Fig. 3C) [24]. Based on these findings, an optimal field strength of 150 V/cm was selected for subsequent experiments. To achieve this field strength using the 30 V voltage from the electrolysis process, the dimensions of the enrichment zone were simulated using COMSOL Multiphysics. The compact design also enabled the integration of 4 parallel enrichment zones within the EEMC, allowing simultaneous and independent enrichment of 4 different samples. Enriched nucleic acids were collected through “M”-shaped channel—aligned with the enrichment zones—that connect to the downstream amplification chamber (Fig. S10).

With the enrichment zone size fixed, we next optimized the flow rate and microfluidic channel configuration. Flow rates ranging from 0 to 75 μl/min were tested. Similar enrichment performance was observed under static conditions and at flow rates of 15 and 30 μl/min, while other flow rates resulted in reduced fluorescence intensity and enrichment efficiency (Fig. S11). Considering both enrichment performance and consistency with prior electrolysis experiments, a flow rate of 30 μl/min was selected as the final flow rate for all subsequent experiments.

Under optimal conditions, pre-lysed samples were enriched using both a commercial kit and the EEMC, followed by qPCR detection (Fig. 3F). Compared to the kit-processed samples, electro-enrichment resulted in lower Ct for all 4 target genes (BAX, CDKN1A, GADD45A, and GAPDH), indicating higher nucleic acid concentration ability. Enrichment efficiency was further evaluated using DNA samples at concentrations ranging from 10^2^ to 10^6^ copies/ml. Both the commercial kit and electro-enrichment significantly enhanced fluorescence intensity compared to the control group; however, the electro-enrichment samples exhibited higher fluorescence intensity (Fig. 3G). The linear regression slope for the electro-enrichment method (0.133) was steeper than that of the commercial kit (0.121), indicating greater sensitivity (Fig. S12).

To further investigate the enrichment efficiency of different nucleic acid concentrations, standard DNA solutions of 10, 50, and 100 ng/μl were tested. The enrichment efficiency of the commercial kit ranged from 88% to 96%, while the electro-enrichment method achieved efficiencies between 92% and 98%, outperforming the commercial kit for all concentrations (Fig. 3H). Additionally, DNA loss rates during the enrichment process were assessed using total DNA amounts of 0.1, 0.5, and 1 μg. Loss was calculated by comparing DNA mass before and after enrichment. The commercial kit exhibited a loss rate ranging from 2% to 12%, whereas electro-enrichment showed a lower loss rate of 1.5% to 7.5% (Fig. 3I). Finally, we evaluated the enrichment consistency across the 4 parallel modules of the EEMC by analyzing BAX, CDKN1A, GADD45A, and GAPDH expression. No significant differences were observed among the 4 modules (*P* ≥ 0.05), confirming the reproducibility and uniformity of the enrichment process (Fig. 3J and K and Fig. S13).

### Optimization and investigation of LAMP based on the EEMC

**Fig. 4.**
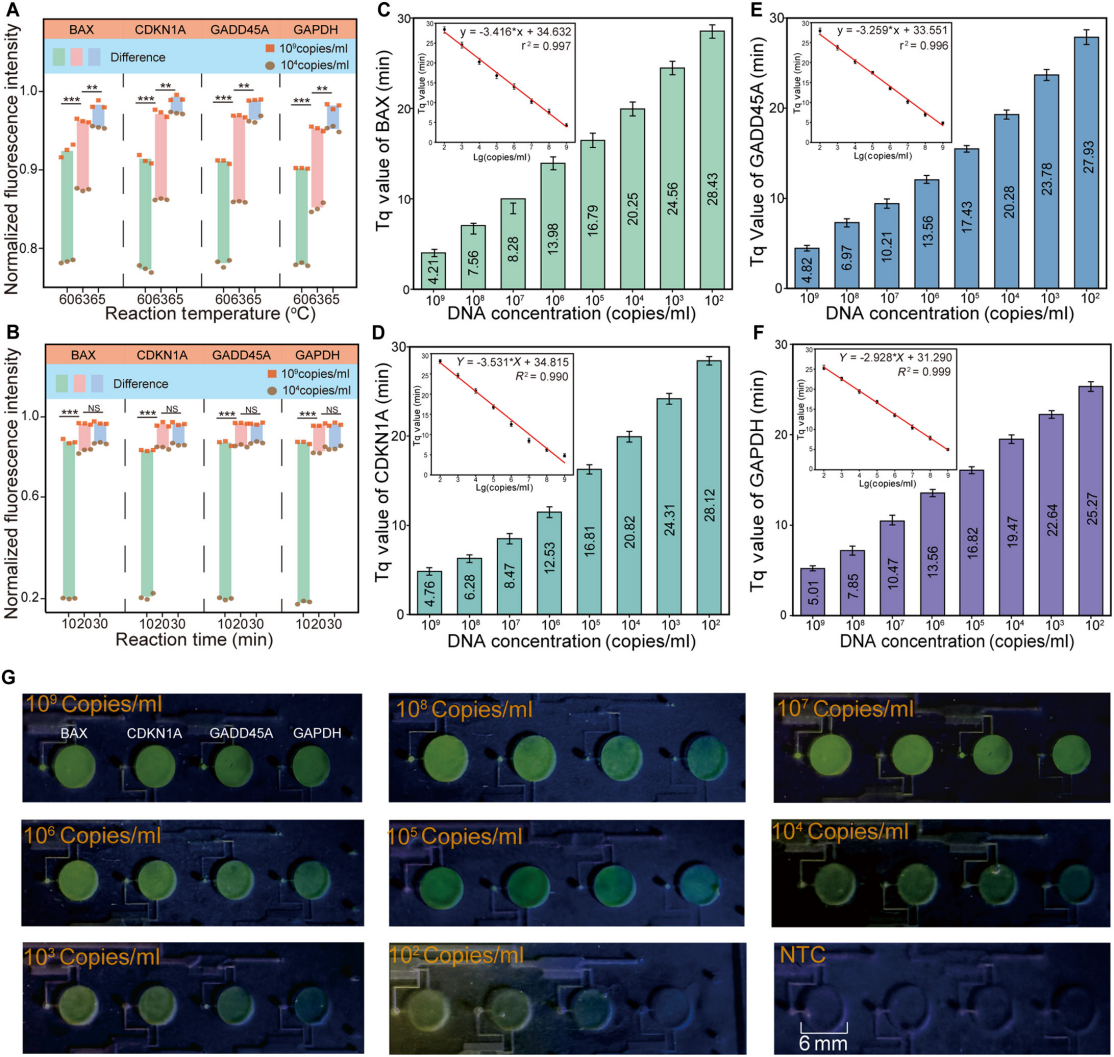
Optimization and detection performance of the EEMC. (A) Optimization of reaction temperature for LAMP. Two template concentrations (10^9^ and 10^4^ copies/ml) were tested at 60, 63, and 65 °C for 15 min (*n* = 3). (B) Optimization of reaction time. Reactions were conducted at 65 °C for 10, 20, and 30 min (*n* = 3). (C to F) Sensitivity testing of LAMP primers targeting BAX (C), CDKN1A (D), GADD45A (E), and GAPDH (F). (G) Representative fluorescent images of the EEMC toward different concentrations of targets from 10^2^ to 10^9^ copies/ml. NTC is the control group, with ddH_2_O added to the system instead of plasmid.

We screened 11 sets of LAMP primers targeting BAX, CDKN1A, GADD45A, and GAPDH using both positive and negative control tests to identify the best-performing primer sets (Fig. S14), with detailed sequences listed in Tables S2 to S5. To optimize the reaction conditions, we first investigated the effects of reaction temperature and time. Reactions were performed at 60, 63, and 65 °C for 20 min. Among these, 65 °C displayed significantly better amplification efficiency and was therefore selected as the optimal reaction temperature (Fig. 4A). Next, we evaluated the impact of reaction time by comparing amplification results at 10, 20, and 30 min. No significant difference was observed between 20 and 30 min, while both were significantly better than the 10-min reaction (Fig. 4B). Considering reaction efficiency and speed, a duration of 20 min was selected for all subsequent LAMP reactions. To assess primer sensitivity, standard plasmid DNA samples ranging from 10^2^ to 10^9^ copies/ml were tested for each of the 4 targets: BAX, CDKN1A, GADD45A, and GAPDH. The results demonstrated that the EEMC-based LAMP assay could reliably detect samples with concentrations of 10^2^ copies/ml and above (Fig. 4C to F). Furthermore, a clear linear relationship was observed between the logarithmic plasmid concentration and the time to quantification value (Tq value) for CDKN1A, GADD45A, and GAPDH, confirming the quantitative capability of the assay (Fig. 4C to F).

It is worth noting that DNA plasmids, due to their high-molecular stability and precise quantification characteristics, allow for the rapid verification of the compatibility of LAMP amplification systems. For mRNA detection, we used 10^2^ to 10^9^ copies/ml RNA standards as templates to evaluate the system’s ability to detect RNA samples. The results show that the detection performance of the system for RNA plasmids is highly consistent with that for DNA plasmids. The limit of detection (LOD) is stable at 10^2^ copies/ml, and the logarithmic concentration and Tq value also show a significant linear relationship, which is comparable to the linear fitting degree of the corresponding DNA plasmids, confirming the quantitative detection ability of the system for RNA samples.

To visually demonstrate the fluorescence output, endpoint images of the LAMP results based on the EEMC were also captured and presented (Fig. 4G). As expected, higher target concentrations corresponded to stronger fluorescence signals (Fig. S15). The fluorescence endpoint image of the RNA plasmid further shows that its fluorescence intensity increases in a gradient with the increase of concentration. Obvious fluorescence signals were observed in RNA samples with RNA concentrations of 10^3^ copies/ml and above. Compared with DNA plasmids of the same concentration, there was no significant difference in signal intensity. The fluorescence of the 10^2^ copies/ml RNA sample was weak and did not exceed the background threshold (Fig. 4E and Fig. S16). These results jointly confirm that the LAMP based on the EEMC not only has excellent detection performance for DNA plasmids but also has high sensitivity and reliable quantitative ability for RNA samples, fully meeting the core requirements of mRNA detection.

### Validation with clinical samples

**Fig. 5.**
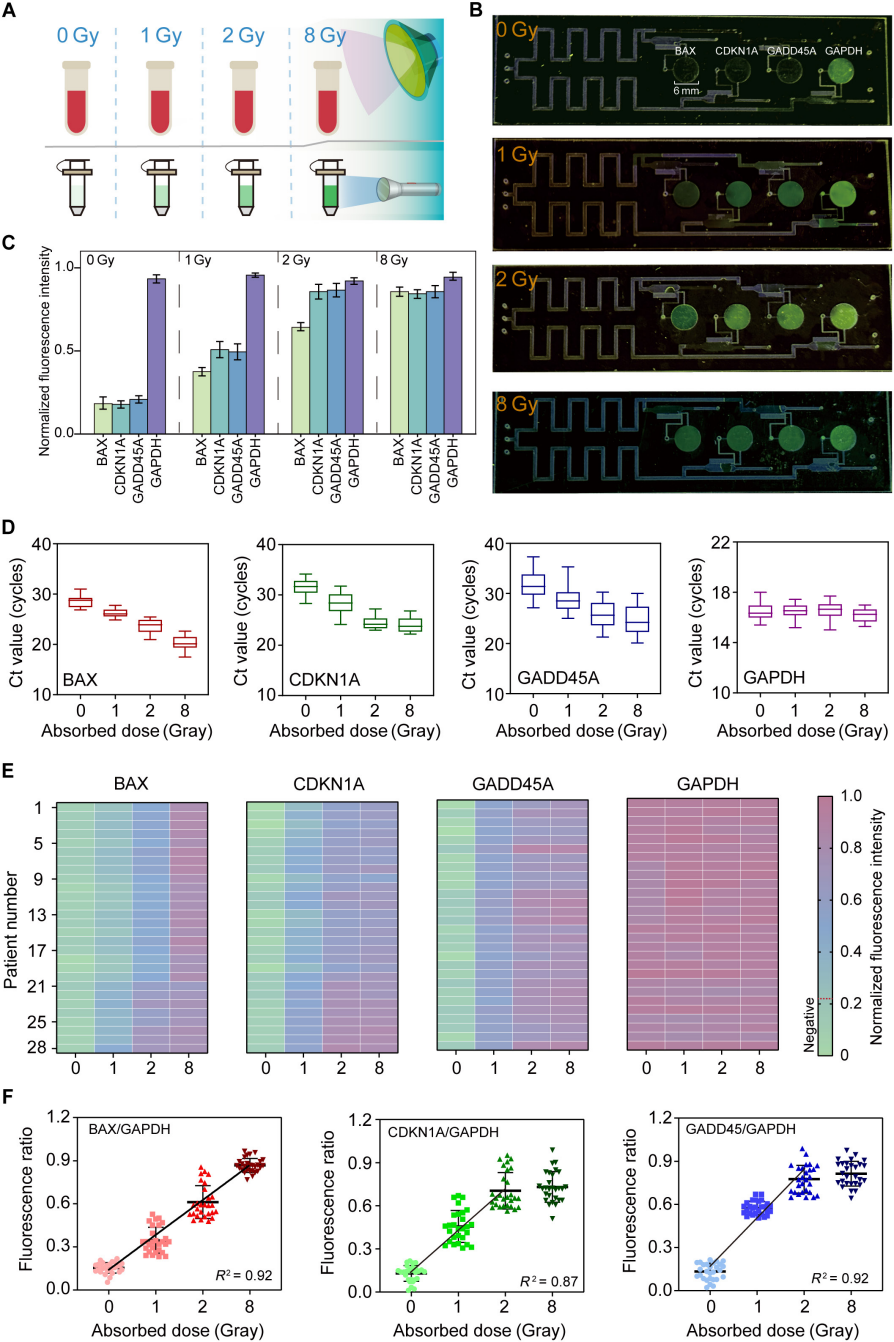
Detection performance of the EEMC toward clinical samples. (A) Schematic representation of blood sample treatment under varying radiation doses, simulating radiation exposure scenarios. (B) Fluorescence intensities of the EEMC toward blood samples under different radiation doses from 0 to 8 Gy. (C) Quantitative and statistical analysis of fluorescence results of different target genes (BAX, CDKN1A, GADD45A, and GAPDH) under different radiation doses. (D) qPCR detection results of 28 human blood samples processed using a commercial kit. (E) Fluorescence detection results of the samples based on the EEMC. (F) Ratios of the target genes (BAX, CDKN1A, and CDKN1A) to the fluorescence signal of GAPDH.

BAX, CDKN1A, and GADD45A are key genes involved in cell cycle regulation, DNA damage response, and apoptosis. Their expression levels vary significantly with different levels of radiation exposure (0, 1, 2, and 8 Gy), providing a means to assess radiation-induced cellular stress (Fig. 5A). Based on fluorescence intensity results from the EEMC (Fig. 5B and C), the expression levels of BAX, CDKN1A, and GADD45A were found to increase with radiation dose, while GAPDH expression remained stable across all conditions, serving as a reliable reference gene. Notably, CDKN1A and GADD45A appeared highly sensitive to radiation compared to BAX, with their expression levels plateaued or slightly declined between 2 and 8 Gy, indicating potential saturation effects at high doses.

This trend was further validated through qPCR analysis of 28 clinical samples processed with the commercial kit across varying radiation doses (Fig. 5D). In unirradiated samples (0 Gy), the expression levels of BAX, CDKN1A, and GADD45A showed low expression levels. Following 1 Gy exposure, Ct values of BAX decreased by 2, while Ct values of CDKN1A and GADD45A decreased by approximately 3. At 2 Gy, all 3 genes exhibited further up-regulation (Ct values of BAX decreased by 5; Ct values of CDKN1A decreased by 7; Ct values of GADD45A decreased by 6). After 8 Gy exposure, BAX expression peaked, whereas CDKN1A and GADD45A expression levels were similar to or slightly higher than those at 2 Gy. Throughout all conditions, GAPDH expression remained stable, confirming its suitability as an internal reference. Consistently, the EEMC, which completes processing within 10 min via electrolytic lysis and ICP enrichment, showed detection results consistent with those of commercial kits. At 0 Gy, BAX, CDKN1A, and GADD45A exhibited low fluorescence intensity; at 1 to 2 Gy, the fluorescence intensity increased gradually; at 8 Gy, BAX peaked while the other genes remained stable, confirming the reliability of EEMC results (Fig. 5E).

To further quantify radiation-induced gene expression changes, the ratios of BAX/GAPDH, CDKN1A/GAPDH, and GADD45A/GAPDH were analyzed. The BAX/GAPDH ratio exhibited a clear linear correlation with radiation dose (Fig. 5F). Similarly, linear relationships were found for CDKN1A/GAPDH and GADD45A/GAPDH ratios when excluding the 8 Gy data point, likely due to biological saturation or negative feedback mechanisms at high radiation doses. Under unexposed conditions, all 3 ratios were <0.2. At low doses, BAX/GAPDH increased to ~0.3, while CDKN1A/GAPDH and GADD45A/GAPDH rose to >0.4. At moderate doses, BAX/GAPDH reached ~0.6, with CDKN1A/GAPDH and GADD45A/GAPDH approaching ~0.7. At high doses, BAX/GAPDH continued to rise to ~0.9, while the other 2 ratios stabilized at ~0.8. These results support the use of gene expression ratios for sensitive radiation dose estimation.

## Conclusion

mRNA detection is widely recognized for its value in disease diagnosis, treatment monitoring, and prognosis evaluation. However, its low abundance, inherent instability, and high susceptibility to RNase degradation pose significant challenges for sample processing and detection [1,2,4]. Therefore, there is a pressing need for innovative analytical technologies that improve the efficiency of mRNA extraction and detection, while offering strong anti-interference capability and reproducibility to support clinical applications.

In this study, we developed and validated an EEMC that integrates rapid electrolysis-based lysis, ICP-based enrichment, and downstream LAMP for mRNA extraction and detection. The EEMC provides a highly efficient and adaptable platform for mRNA analysis. Its speed and operational simplicity offer significant advantages over conventional methods. We selected 3 representative mRNA biomarkers, including BAX, CDKN1A, and GADD45A to evaluate the system’s utility, which are known to be related with nuclear radiation [8,14,15].

Compared to traditional techniques, the EEMC offers several notable advantages. It eliminates the need for chemical-based extraction methods and simplifies the overall workflow, significantly reducing sample processing time and the risk of mRNA degradation or interference. From a cost perspective, it dramatically lowers per-sample expenses—from approximately $2 using commercial kits to under $0.50—enhancing its feasibility for large-scale clinical screening. Future improvements may aim to further enhance sensitivity and specificity through precise fluid control and modular chip designs.

In summary, the EEMC presents a powerful, rapid, cost-effective, and highly sensitive solution for integrated mRNA detection. Its unique combination of electrolysis, enrichment, and amplification technologies paves the way for rapid, on-site molecular diagnostics and offers valuable support for advancing precision medicine and personalized healthcare.

## Materials and Methods

### Materials and reagents

The microfluidic chip was fabricated using acrylic plates through computer numerical control (CNC) machining (Beijing Hongyang Chensheng Technology Co., Ltd., China). The power supply system adopted the MS-1003DS power supply from Maisheng Company (output voltage range: 0 to 99.00 V, current range: 0 to 3.00 A). The sample delivery device used was the TYD02 syringe pump from Leadfluid Company (China). The key material for the electro-enrichment module, a 20 wt% Nafion perfluorinated resin solution, was purchased from Shanghai Macklin Biochemical Technology Co., Ltd. (China), to construct the cation-selective permeable membrane.

A549 cells were purchased from the American Type Culture Collection (ATCC) cell bank and were used for optimizing the conditions of electrolysis and enrichment. For cell culture, RPMI1640 medium and fetal bovine serum from Gibco Company were used; phosphate-buffered saline (PBS, pH 7.4) was provided by Beijing Solarbio Science & Technology Co., Ltd. An Olympus CKX53 microscope (Tokyo, Japan) was used to observe the state of the cells. The Calcein AM dye for live cell staining was purchased from Sangon Biotech (Shanghai, China), and the PI dye for dead cell staining was purchased from Yisheng Biotech Co., Ltd. (Shanghai, China).

Human blood samples were provided by the Chinese People’s Liberation Army General Hospital. Commercial mRNA extraction and purification were performed following the standard procedures of the Molpure Blood RNA Kit from Yeasen Biotechnology Company. The concentration and purity of the DNA samples were detected using the SPARK microplate reader from TECAN Company.

The synthesis of primers and plasmids was provided by Hippo Biotechnology Co., Ltd. (Shanghai, China) (Tables S2 to S5). The HiScriptIII All-in-one RT SuperMix Perfect for qPCR kit for mRNA reverse transcription and the Taq Pro Universal SYBR qPCR Master Mix kit for qPCR amplification were both purchased from Vazyme Biotech (Nanjing, China). The Warm Start LAMP 2× Master Mix reagent for LAMP detection was purchased from New England Biolabs (Ipswich, MA, USA). All real-time qPCR and LAMP reactions were completed on the CFX96TM Optics Module (BIO-RAD, Singapore) thermal cycling instrument. The LAMP reaction inside the chip was heated at a constant temperature in a water bath. The fluorescence signals emitted by the amplification products in the amplification region were detected using a handheld blue light lamp (Luyor, 3260RB, Shanghai, China), and photos were taken with an iPhone 16 pro.

### Design and fabrication of the chip

The chip structure was modeled using the AutoCAD 2023 and fabricated through CNC machining. The overall shape of the chip is rectangular, with dimensions of 90 mm × 25 mm × 2 mm, and it is composed of 2 functional modules, the upper layer and the lower layer.

The upper layer is the microfluidic functional layer, integrating 3 core functional areas: electrolysis, electro-enrichment, and fluorescence detection. Among them, the electrolysis area contains 2 parallel channels, and the size of a single channel is 50 mm × 0.8 mm × 0.3 mm (length × width × height); the electro-enrichment area is composed of 4 parallel enrichment units with dimensions of 4 mm × 2 mm × 0.3 mm, which can achieve multiple nucleic acid enrichment and synchronous detection. In each enrichment unit, 29 arrayed channels (1 mm × 0.1 mm × 0.1 mm) are arranged near the negative electrode side, which are used to fix the Nafion perfluorinated resin solution to construct an ion-selective membrane. After the nucleic acids are electro-enriched, they are transported to the detection area through an “M”-shaped channel. The detection area is composed of 4 independent cylindrical chambers (radius 3 mm, depth 1 mm). Each chamber is preloaded with lyophilized primers and LAMP reagents, enabling the immediate amplification and detection of nucleic acids.

After chip processing, surface cleaning was carried out using 75% ethanol and sterile water, and then functional modification was performed. For the electro-rich area, 2 μl of 20 wt% Nafion solution was dropped into the array channel, evenly coated and cured at room temperature to form a solid ion-selective permeable membrane perpendicular to the channel direction. For the detection area, 20 μl of the LAMP reaction system mixture (containing specific primers) was added to each chamber respectively, to form a pre-encapsulated reaction unit after freeze-drying treatment.

The lower layer is the electrode fixing layer, which is designed with positive and negative electrode grooves (length, 90 mm; width, 0.8 mm; and depth, 1 mm) running through the electrolysis and electro-enrichment areas. A stable electric field generating device is constructed by embedding wires.

Then, the upper and lower layers of the chip were bonded using ultraviolet (UV) glue (Ergo 8500, Switzerland), which was cured under a 365-nm UV lamp (Luyor-3650, Shanghai Luyor Instrument Co., Ltd.) for 60 s. Finally, a microfluidic chip system integrating nucleic acid lysis, enrichment, and amplification is formed, which can achieve automated nucleic acid detection under stable electric field conditions.

### Chip operation procedure

The EEMC has the ability to perform nucleic acid detection for the whole process of a 1.2-ml liquid sample, including lysis, enrichment, and amplification detection. The sample is transported into the chip by a syringe pump. After applying a direct current electric field in the electrolysis area, the high-intensity electric field can efficiently rupture the cell membrane within 10 s, prompting the rapid release of intracellular mRNA. The lysis products then flow into 4 parallel enrichment modules. Under the synergistic effect of the lateral liquid driving force and the vertical ICP enrichment force, mRNA molecules are directionally enriched to form an ion flow, which is transported to the preset reaction chambers through the “M”-shaped channel.

Each reaction chamber is preloaded with lyophilized detection reagents, including specific primers, the LAMP buffer system, reverse transcriptase, and other essential reaction components. When the enriched mRNA is fully reconstituted and mixed with the lyophilized reagents, the chip is placed in a constant temperature water bath at 65 °C to initiate the isothermal amplification reaction. After the reaction for 20 min, a handheld UV excitation device is used to perform fluorescence detection on the reaction chambers, and the rapid detection of the target nucleic acid is achieved by observing the intensity of the fluorescence signal.

### Simulation of the liquid flow direction inside the chip

A simulation study on fluid dynamics inside the chip was carried out based on the COMSOL Multiphysics platform. A 3-dimensional model of the microchannels of the chip was constructed using AutoCAD according to the actual dimensions (90 mm × 25 mm × 2 mm) and imported into the simulation environment. The laminar flow (single-phase flow [SPF]) physical field module was selected. The inlet boundary condition was set as the volumetric flow rate (*Q* = 120 μl/min), the outlet was set as a pressure outlet, and the wall surface of the flow channel was defined as a no-slip boundary. According to the continuity Eqs. 1 and 2:

A=wh (1)

$$v_{\text{inlet}}=\frac{Q}{A}$$ (2)

where *A* is the cross-sectional area, and *w* and *h* correspond to the width (0.8 mm) and height (0.3 mm) of the flow channel, respectively. The calculated average flow velocity at the inlet was 8.33 mm/s. The fluid medium selected was a 1× PBS solution, and its density *ρ* and dynamic viscosity *μ* were set as constants. Numerical solutions were carried out based on the incompressible Navier–Stokes equation (Eq. 3) and the continuity equation (Eq. 4):

$$\rho \left(\frac{\partial v}{\partial t}+v∙\nabla v\right)=-\nabla p+\mu \nabla^{2}v+F$$ (3)

∇v=0 (4)

where *v* is the velocity field, *p* is the pressure, and *F* is the external force (*F* = 0 in this study). The stress tensor τ is defined by the velocity gradient tensor ∇*v* (Eq. 5):

$$\tau =\mu \left(\nabla v+{\left(\nabla v\right)}^{\tau }\right)$$ (5)

The finite element method was used to discretely solve the above equations to obtain the velocity distribution in the flow channels of the chip. The split flow rates of each functional area were calculated through the surface integral (Eq. 6):

$$Q_{i}=\int_{S_{i}}v∙\mathrm{ndA}$$ (6)

The simulation results showed that, after the liquid entered the lysis area, it was evenly divided into 2 streams (each 60 μl/min) in the electrolysis zone; when flowing from the lysis area to the 4 parallel enrichment area, the split flow rates of the upper half of the enrichment units were 32 and 28 μl/min, respectively, and those of the lower half units were both 30 μl/min, providing a theoretical basis for the structural optimization of the functional areas of the chip.

### Simulation of the electric potential and electric field inside the chip

Based on the COMSOL Multiphysics platform, a multi-physics field coupling model of the electric field and fluid-matter transport inside the chip was constructed. A 2-dimensional electric current physical field was established, and the material properties of the PBS solution (conductivity *σ* = 0.65 S/m, relative permittivity *ϵ*_r_ = 76) and the gold electrode (conductivity *σ* = 4.5×10^7^ S/m) were defined. The electric field distributions in the electrolysis area (10 mm × 0.8 mm × 0.3 mm) and the electro-enrichment area (4 mm × 2 mm × 0.3 mm × 4 array) were solved by Poisson’s equation: where *ϕ* is the electric potential, *ϵ* = *ϵ*_0_*ϵ*_r_ is the permittivity, and *ρ*_e_ is the charge density. The boundary conditions were set as follows:

1. Dirichlet boundary: An electric potential of *V*_0_ = 30 V was applied to the positive electrode, and the negative electrode was grounded (*V* = 0 V).
2. Neumann boundary: n∙ϵ∇∅=0 (insulated boundary).
3. Current continuity equation: ∇∙J = 0, J=σE.

A steady-state solver was used to calculate the electric potential and electric field distributions, the electric field strength was obtained based on this, and the effect of the electric field was evaluated through the formula of the electric potential energy density per unit volume.

### Simulation of the electro-enrichment function

The laminar flow (SPF) and transport of diluted species (TDS) physical fields were coupled to construct a 2-dimensional fluid-matter transport model. The enrichment region was set as a rectangle with dimensions of 4 mm × 2 mm. Positive and negative electrodes were arranged at the top and bottom, and the surface of the negative electrode was modified with a Nafion cation exchange membrane.

In the SPF module, according to the above flow channel simulation, the inlet was set on the left side of the region, the inlet flow rate *Q* = 30 μl/min (corresponding to a flow velocity *v* = 2.08 mm/s), and the outlet pressure *P* = 0 Pa.

In the TDS module, the molecular mobility was calculated according to the Nernst–Einstein relationship:

$$\mu_{j}=\frac{z_{j}FD_{j}}{\mathrm{RT}}$$ (7)

The Nernst–Planck equation was used to describe the transport of charged molecules:

$$\frac{\partial c}{\partial t}=\nabla ∙\left(D\nabla c-\mathrm{vc}+\text{μzFc}\nabla \emptyset \right)$$ (8)

The initial condition was *c* = *c*_0_ (uniform distribution). The boundary conditions were a constant inlet concentration *c*_0_ = 1 mol/m^3^ and an outlet flux of 0; the DNA molecular transmittance of the Nafion membrane was set to 0. The physics-controlled mesh was used for meshing to refine the Nafion membrane region. A time-dependent solver was used, and the time range was set from *t*_S_ = 0 s to *t*_E_ = 10 s, with a time interval 0.1 s. The dynamic enrichment process of negatively charged nucleic acid molecules under the action of the electric field, convection, and diffusion was simulated to obtain the molecular concentration distribution and migration paths.

### Optimization of electrical lysis conditions

The key parameters influencing electrical lysis include voltage and lysis duration. A total of 1×10^6^ cells were resuspended in 200 μl of 1× PBS buffer and infused into the microfluidic chip via a syringe pump at a flow rate of 120 μl/min. Based on preliminary flow distribution simulations, the actual flow rate reaching the lysis zone was 60 μl/min. After complete sample loading into the lysis zone, a direct current (DC) power supply was applied with voltage gradients of 10, 20, 30, 40, and 50V, combined with lysis duration gradients of 5, 10, 15, 20, and 25 s, constituting 25 experimental groups.

Post-lysis samples were dual-stained with Calcein-AM and PI. Utilizing the property that Calcein-AM exclusively enters live cells and is hydrolyzed by esterases to produce green fluorescence, while PI penetrates dead cell membranes and binds to nucleic acids emitting red fluorescence, images were acquired using an Olympus CKX53 inverted fluorescence microscope. Quantitative analysis of viable cell regions was performed using ImageJ software to calculate cell mortality rates, thereby evaluating the extent of cell lysis.

Considering that released mRNA from lysed cells is essential for downstream detection, post-lysis solutions were collected for PCR amplification, targeting the GAPDH gene as an internal reference, and the release efficiency of mRNA was compared by analyzing Ct values across different lysis conditions. The Ct value exhibits an inverse correlation with initial template quantity—lower Ct values indicate more complete cell lysis and higher mRNA abundance.

### Evaluation of the effect of electrical lysis method

Based on the optimized parameters of electrolytic lysis, a systematic comparative analysis was conducted on the nucleic acids extracted by the electrolytic lysis technology and the commercial RNA extraction kit (Yeasen Molpure Blood RNA Kit) from 3 aspects: fragment integrity, gene amplification effect, and detection sensitivity.

To ensure the comparability of the comparison results between the 2 extraction methods, we ensured the consistency of the RNA elution volume. For commercial kits, RNA was eluted to 20 μl of RNase-free water. For RNA collected after electrolytic lysis, it was also adjusted to a final volume of 20 μl with RNase-free water, consistently matching the kit’s elution volume.

The fragment distribution of the nucleic acid samples extracted by the 2 methods was characterized by capillary electrophoresis technology (Agilent 2100 Bioanalyzer, Agilent Technologies). To avoid bias in fragment integrity comparison caused by inconsistent sample loading, 100 ng of RNA (normalized based on the quantified concentration) from each extraction method was loaded for electrophoresis. By analyzing the electrophoresis profiles of nucleic acid fragments of different lengths, the influence of the electrolysis process on the integrity of nucleic acids and the selective collection ability of fragments were evaluated, providing experimental basis at the molecular level for optimizing the lysis conditions.

For the gene amplification effect of the extracted nucleic acids, taking the radiation responsive-related marker genes BAX, CDKN1A, and GADD45A and the internal reference gene GAPDH as the targets, the nucleic acid samples extracted by the 2 methods were amplified by qPCR technology. To ensure uniform RNA input for qPCR, both the kit-extracted RNA and EEMC-enriched mRNA used 2 μl of template for each qPCR reaction, guaranteeing that the same proportion of total RNA is analyzed across groups. By comparing the differences in Ct values and analyzing the influence of different extraction methods on the effect of gene amplification, the performance of electrolysis technology in actual molecular detection is revealed.

For the detection sensitivity, cell concentration gradient (10^1^ to 10^7^ cells) samples were constructed, and nucleic acid extraction and subsequent qPCR amplification were carried out respectively by electrolysis technology and commercial methods. By comparing the detected Ct values, the extraction and detection sensitivities of the 2 methods for different initial sample sizes were evaluated. Meanwhile, based on the ratio of the target genes (BAX, CDKN1A, and GADD45A) to the internal reference genes (GAPDH), the expression levels of the target genes relative to the internal reference genes were calculated, and the influence of the electrolysis process on the expression of the target genes was analyzed.

### Optimization of electro-enrichment conditions

According to the dICP electro-enrichment theory, the enrichment behavior of nucleic acid molecules within microfluidic chips is driven by multiple forces. In the vertical direction, it is driven by electrophoretic force and electroosmotic force, while in the horizontal direction, it is affected by fluid driving force. The final enrichment sites are jointly determined by flow velocity and electric field intensity. Under the condition of a fixed flow rate of 120 μl/min (based on the previous fluid simulation results of the flow channel), the influence mechanism on nucleic acid enrichment sites was explored by regulating the parameters of the electric field intensity.

FAM-labeled single-stranded DNA (FAM-ssDNA; 10^4^ pM) was used as the tracer, and electric field intensity gradients of 50, 100, 150, 200, and 250 V/cm were optimized. The electro-enrichment region was observed in real time by the fluorescence microscope, and the fluorescence distribution of FAM-ssDNA under different electric field conditions was recorded and quantitatively analyzed by ImageJ software. Through the statistics of fluorescence values and the spatial distribution position, the relationships between the electric field intensity and the nucleic acid enrichment effect and enrichment sites were constructed.

### Evaluation of electro-enrichment effect

To evaluate the enrichment efficiency and amplification performance, standard plasmids of BAX, CDKN1A, GADD45A, and GAPDH genes were synthesized, and samples with a concentration gradient of 10^2^ to 10^9^ copies/ml were prepared. Plasmid DNA is more stable than mRNA and allows precise control of concentration, making it ideal for optimizing critical parameters like electric field intensity and LAMP, avoiding RNA degradation-related artifacts during optimization.

The 10^5^ copies/ml standard plasmid was treated respectively by electro-enrichment technology and a Yeasen nucleic acid filter column. After the enrichment products were amplified by qPCR, the effects of different techniques on nucleic acid enrichment and amplification efficiency were quantified by comparing the circulation thresholds (Ct values) before enrichment (control group) and after treatment by the 2 methods.

For the enrichment sensitivity of plasmids with different concentrations, FAM-ssDNA plasmids with a concentration gradient of 10^2^ to 10^6^ copies/ml were used. After electro-enrichment treatment and silicon-based filter column treatment, images were collected using the fluorescence microscope. The fluorescence intensity of the samples before and after enrichment was quantitatively analyzed by ImageJ software, and the background error was eliminated through normalization processing. The sensitivity response trends of the 2 methods at different concentrations were compared.

To quantitatively analyze the enrichment efficiency of nucleic acids at different concentrations, DNA plasmid samples of 1, 5, and 10 ng/μl were prepared. After being treated by 2 enrichment methods, the concentration of the enrichment products was determined by TECAN microplate reader. Based on the formula calculation:

$$\text{Concentration rate}=\frac{\text{Actual enrichment concentration}}{\text{Theoretical enrichment concentration}}$$ (9)

The absolute enrichment efficiency of the electro-enrichment technology and the silicon-based filter column was compared.

For the loss rate of DNA during the enrichment process, DNA plasmid samples of 100, 500, and 1,000 ng were prepared. The content of DNA samples before and after enrichment was measured by the microplate reader. The DNA loss rates of the 2 methods during the enrichment process was evaluated using the following formula:

$$\begin{array}{c} \begin{array}{c} \begin{array}{c} \mathrm{DNA}\;\text{loss rate}=\frac{\text{Theoretical enrichment concentration}-\text{Actual enrichment concentration}}{\text{Theoretical enrichment concentration}} \end{array} \end{array} \end{array}$$ (10)

For the consistency verification of the 4 parallel enrichment regions inside the chip, the BAX, CDKN1A, GADD45A, and GAPDH standard plasmids of 10^5^ copies/ml were mixed and respectively introduced into the 4 parallel enrichment regions upstream and downstream of the micro-nano chip. After enrichment, qPCR amplification was performed on the products in each region. By comparing the intra-group differences in the gene amplification results in the upstream/downstream regions, the uniformity and reliability of the multi-channel enrichment performance of the chip were verified, ensuring the uniformity of the sample distribution among the parallel modules of the EEMC.

### LAMP reaction system optimization

Multiple sets of LAMP primers were designed respectively for the BAX, CDKN1A, GADD45A, and GAPDH genes. Through positive and negative sample tests, the optimal primer combination for each target gene was screened out based on the amplification effect to ensure the specificity and effectiveness of the primers. In order to optimize the reaction temperature, plasmids with concentrations of 10^4^ and 10^9^ copies/ml were selected as templates. The LAMP reaction was carried out under 3 temperature conditions of 60, 63, and 65 °C, respectively, and the reaction time was fixed at 20 min. Meanwhile, under the condition of a reaction temperature of 65 °C, the reaction time gradients were set at 10, 20, and 30 min to optimize the reaction time. After the reaction was completed, the fluorescence intensities of the BAX, CDKN1A, GADD45A, and GAPDH target systems were normalized. By comparing the fluorescence intensities at different reaction temperatures and times, the influence on the LAMP reaction results was analyzed to determine the optimal reaction conditions.

For the sensitivity evaluation of different targets, DNA plasmids and RNA plasmids with a concentration range of 10^2^ to 10^9^ copies/ml were used as templates. LAMP amplification was performed on the real-time qPCR instrument, and the Tq value (LAMP Ct) during the amplification process was recorded to evaluate the detection sensitivity of different targets on the real-time qPCR platform. Similarly, LAMP reactions of different concentrations were carried out in the reaction chamber of the micro-nano chip. After the reaction was completed, the fluorescence results were observed using a handheld UV lamp to visually evaluate the detection sensitivity of different targets on the chip platform.

### Clinical blood sample processing and testing

Blood samples (2 ml) were obtained from hospitals and subjected to radiation treatment at different doses (0, 1, 2, and 8 Gy). White blood cells were extracted by density gradient centrifugation and resuspended in 1× PBS buffer. Subsequently, the samples were evenly divided into 2 parts for chip detection and commercial kit detection respectively, and the expression of BAX, CDKN1A, and GADD45A genes under different radiation doses was analyzed.

Total RNA was extracted from white blood cell samples using the Molpure Blood RNA Kit from Yeasen Biotechnology in accordance with the standard operating procedures and then qPCR amplification was conducted. The Ct value of the amplification was recorded by a real-time fluorescence qPCR instrument, which was used as a quantitative indicator of the gene expression level.

The white blood cell samples resuspended in PBS buffer were directly injected into the micro-nano chip. The electrolysis module achieved efficient rupture of the cell membrane in a short time and released the nucleic acid substances within the cells. Subsequently, nucleic acid molecules were rapidly enriched in the electro-enrichment region to enhance the detection sensitivity. Ultimately, the enriched nucleic acid samples underwent LAMP reactions in the reaction chambers. The amplification products were labeled with fluorescent dyes to achieve visual detection of gene expression. The fluorescence images were analyzed using ImageJ software, and the fluorescence intensity was normalized to eliminate experimental errors and background interferences, ensuring the accuracy and reliability of the detection results.

### Real-time PCR

The qPCR reaction was carried out using the Taq Pro Universal SYBR qPCR Master Mix kit. According to the instruction manual, the 20-μl reaction system was configured. Each reaction contained 10 μl of qPCR Master Mix, 0.4 μl of forward and reverse primers each, 2 μl of template DNA, and 7.2 μl of ddH_2_O.

### LAMP

The LAMP reaction was carried out using the Warm Start LAMP 2× Master Mix kit. The 20-μl reaction system was configured according to the instruction manual. Each reaction contained 12.5 μl of LAMP buffer, 1.4 μl of dNTPs,1 μl of Bst DNA polymerase, 0.5 μl of reverse transcriptase, 2.5 μl of primer mixture, and 0.25 μl of Sybr Green. The prepared solution was added dropwise to the reaction zone of the chip and freeze-drying treatment was carried out. The LAMP reaction (preloaded in detection chambers) uses reverse transcriptase to convert mRNA to cDNA, followed by isothermal amplification. DNA (genomic or contaminating) cannot be reverse transcribed and thus does not contribute to amplification.

### Data analysis

The standard deviation in this article was calculated from the data of 3 independent trials. Fluorescence comparison data were normalized to the minimum and maximum. For statistical analysis, the *P* value was tested by *t* test. All data were analyzed and presented using Origin software. The image results were analyzed using the ImageJ software.

## Data Availability

All data generated or analyzed in the paper are present in the paper and/or the Supplementary Materials.
