# Supplementary material for: An Integrated Electrolysis-Enrichment Microchip for Ultra-Rapid and Sensitive mRNA Detection
Source: Research (Wash D C). 2025 Oct 9;8:0928. doi: 10.34133/research.0928 (PMC12696694; doi:10.34133/research.0928)
Supplement: Supplementary 1 — Figs. S1 to S15 Tables S1 to S5 Movie S1 [file research.0928.f1.zip › supporting datas-Revise.docx]

SUPPLEMENTARY MATERIALS

Title

An Integrated Electrolysis-Enrichment Microchip for Ultra-Rapid and Sensitive mRNA Detection

**Authors**

Long Cheng^1†^, Zhiying Wang^2,3†^, Chengbao Wu^1†^, Feng Liu^2,3^, Hui Li^4^, Yunke Feng^4^, Xi Chen^5*^, Xinxin Hang^2,3^, Yu Zeng^2,3^, Wei Mu^2,6*^, Yuhao Zhou^2,3^, Liye Liu^1^, Lingqian Chang^2,3*^, Qiaowei Liu^4*^, Yi Hu^4,7*^, Yang Wang^2,6*†^

**Affiliations**

^1^ School of Biomedical Engineering, Anhui Medical University, Hefei, 230032, China

^2^ Key Laboratory of Biomechanics and Mechanobiology, Ministry of Education, Beijing Advanced Innovation Center for Biomedical Engineering, Beihang University, Beijing, 100191, China

^3^ School of Biological Science and Medical Engineering, Beihang University, Beijing, 100191, China

^4^ Department of Oncology, the Fifth Medical Center, Chinese PLA General Hospital, Beijing, 100071, China

^5^ Department of Ophthalmology, Beijing Friendship Hospital, Capital Medical University, Beijing, 100050, China

^6^ School of Engineering Medicine, Beihang University, Beijing, 100191, China

^7^ Medical School of Chinese PLA, Beijing, 100853, China

* Address correspondence to: [dr_liuqiaowei@126.com](mailto:dr_liuqiaowei@126.com) (Q.L.); [huyi301zlxb@sina.com](mailto:(huyi301zlxb@sina.com) (Y.H.); [lingqianchang@buaa.edu.cn](mailto:lingqianchang@buaa.edu.cn) (L.C.), [wangyang2022@buaa.edu.cn](mailto:wangyang2022@buaa.edu.cn) (Y.W.)

† These authors contributed equally to this work.


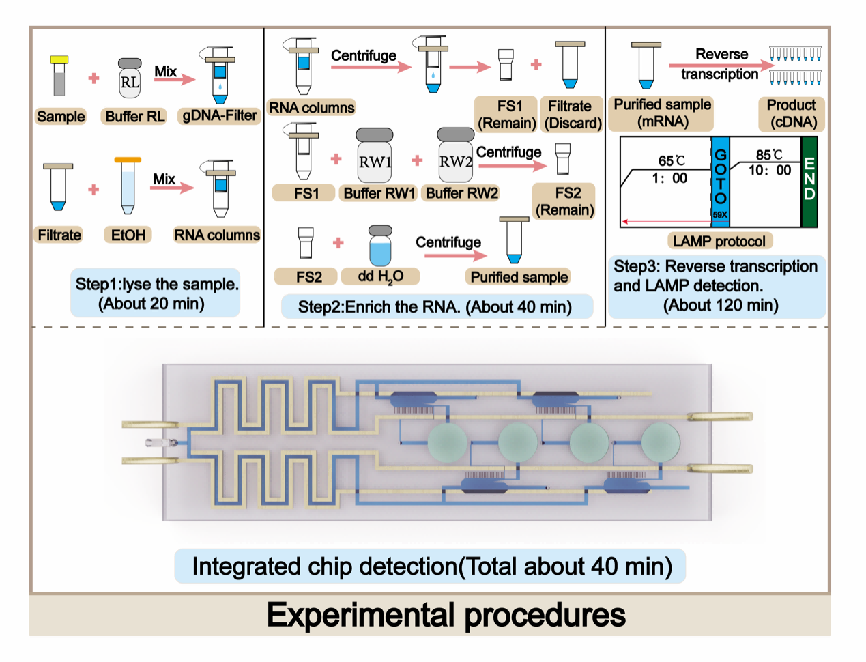


**Supplementary Fig. 1 | Comparison of Detection Processes: Commercial Kit vs. Integrated Chip.** Comparison of the sample detection process using a commercial kit versus the integrated chip.Commercial Kit Process: The detection involves three distinct steps—lysis, purification, and detection—each performed sequentially. Reagent-based lysis takes approximately 20 minutes, purification requires about 40 minutes, and detection takes around 2 hours. The entire process is complex, time-consuming, and relies heavily on extensive reagents and specialized equipment. Integrated Chip Process: The sample is simply introduced into the chip, power is applied, and once the detection chamber is filled, the power is turned off. A constant-temperature heating device is then activated, and fluorescence is observed after the reaction completes. The entire chip-based detection process takes approximately 40 minutes, offering simplicity, portability, and low cost.


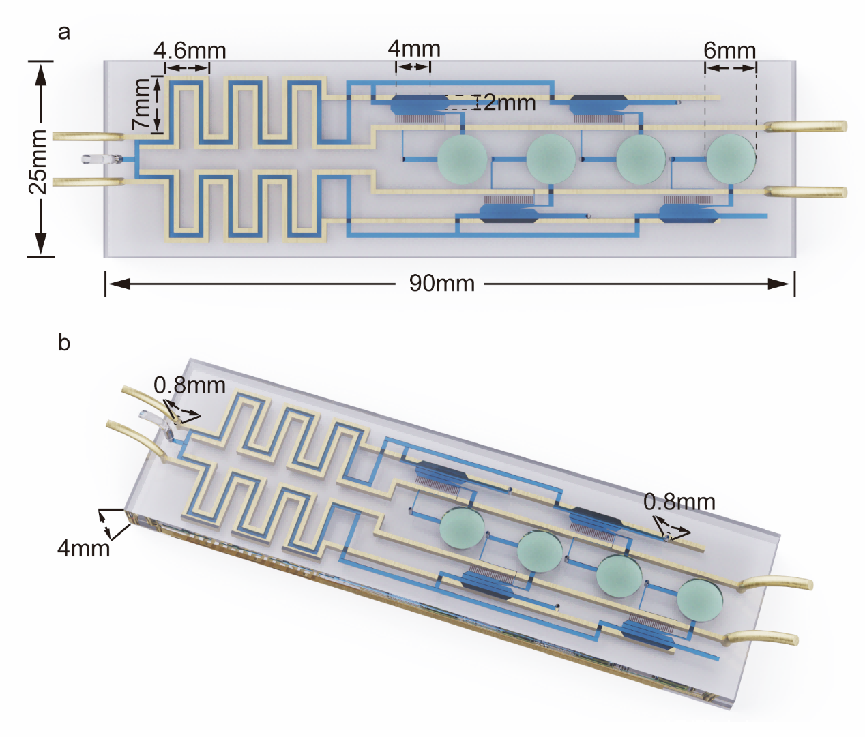


**Supplementary Fig. 2 | Dimensions of the Integrated Chip Design.** Overall dimensions and structure of the integrated chip. Chip Composition: The chip consists of two acrylic plates, each measuring 90 mm in length, 25 mm in width, and 2 mm in height.Upper Plate: The flow channel region, designed for sample flow and modification with lyophilized reagents.Lower Plate: The electrode region, used to secure gold electrodes. The outer electrodes (two wires on the left in the diagram) connect to the positive terminal of the power supply, while the inner electrodes (two wires on the right) connect to the negative terminal.Ports: All inlet and outlet ports have a diameter of 0.8 mm.Electro-Lysis Zone: Divided into upper and lower sections, each with a width of 0.8 mm, a total length of 60 mm, and a channel height of 0.3 mm. Electro-Enrichment Zone: Comprises four modules, each with a flow channel measuring 4 mm in length, 2 mm in width, and 0.3 mm in height. These channels split into three directions: the upper and lower sections lead to outlets, while the middle section directs flow to the detection zone. Detection Zone: Consists of a cylindrical structure with a diameter of 6 mm and a height of 1.5 mm, pre-modified with lyophilized reagents required for the reaction.

**
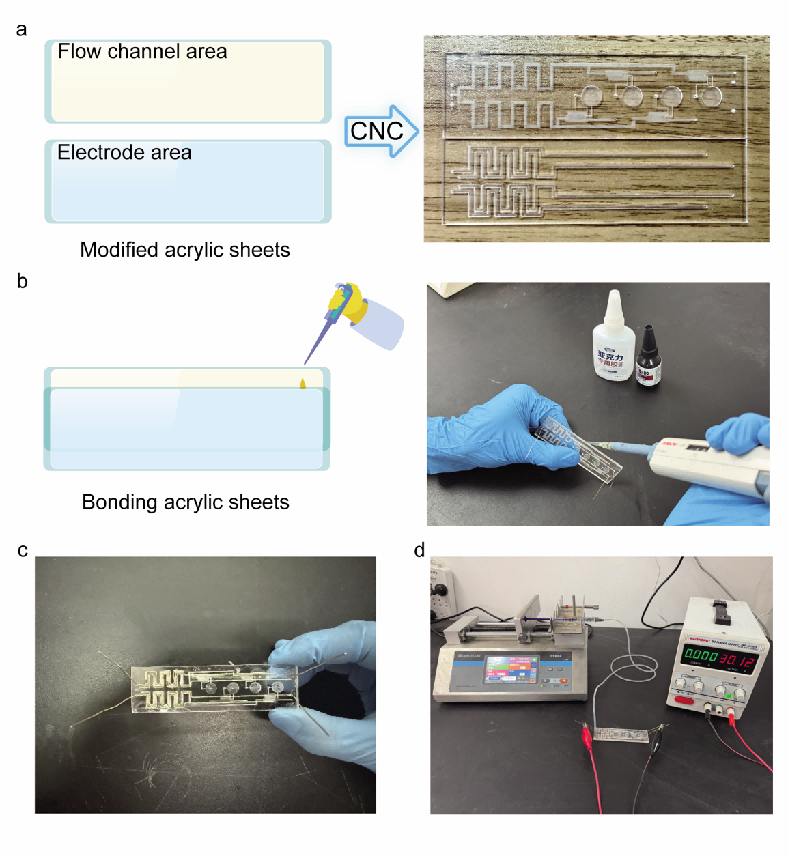
**

**Supplementary Fig. 3 | Chip Fabrication and Detection Platform Setup.** (a) CNC machining of acrylic plates. The acrylic plates are processed using CNC machining to carve grooves on corresponding sections. The grooves on the upper plate serve as flow channels, while those on the lower plate are designed to secure electrodes. (b) Bonding of upper and lower plates with acrylic adhesive. The processed plates (modified with lyophilized reagents and fixed electrodes) are aligned and bonded. Acrylic glue is slowly applied along the edges using a pipette, allowing the adhesive to naturally fill the interior of the chip. A continuous ring of glue is applied around the perimeter, followed by gentle pressing to ensure even distribution. Bonding is completed after waiting for one minute. (c) Physical image of the fully bonded integrated chip. (d) Chip detection platform setup.Leftmost component: A syringe pump, which holds a syringe and injects the sample into the chip via a specialized microfluidic needle and tubing. Middle component: The integrated chip itself.Rightmost component: A DC power supply, with the positive terminal connected to the two left electrodes of the chip and the negative terminal connected to the two right electrodes.


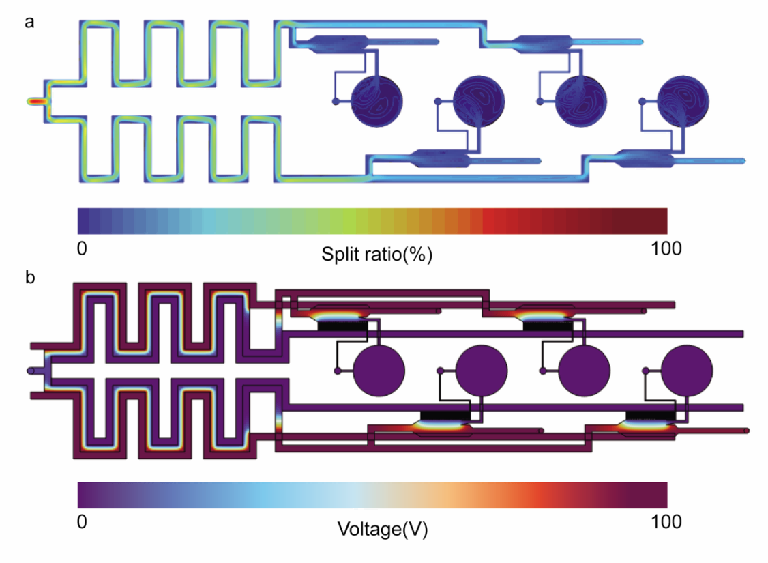


**Supplementary Fig. 4 | Simulation of Electric Potential and Flow Velocity in the Chip.** (a) Simulation of fluid flow velocity distribution across functional zones using COMSOL Multiphysics. The initial input flow rate is 120 μL/min. Through analysis: The electro-lysis zone receives a split flow of approximately 60 μL/min. Each module in the electro-enrichment zone has a split flow of about 30 $\mu L/min.$ The flow from each enrichment module through its channel to the detection zones is approximately 3 μL/min. Based on this flow simulation, the sample volume required to fill the detection zone can be estimated. The detection zone has a capacity of about 30 μL, and with a flow rate allocation requiring 10 minutes to fill, the total sample volume processed per run is approximately 1.2 mL. (b) Simulation of the overall electric potential distribution in the chip using COMSOL Multiphysics. This ensures the rationality of the circuit layout within the chip, preventing issues such as short circuits or open circuits, and confirming that all functional zones are electrically connected.


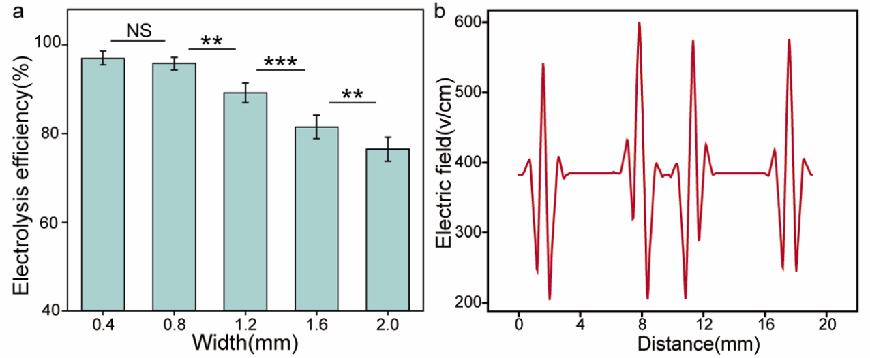


**Supplementary Fig. 5 | Investigation of Electric Field Strength in the Electrolysis Region.** (a) Different widths alter the electric field strength and distribution between the two electrodes, with results showing the electrolysis efficiency of the electrolysis region designed with varying width dimensions. (b) Data analysis of the electric field strength simulation results was performed using ImageJ. Based on the electric field strength distribution in the electrolysis region shown in Figure 1a, the electric field strength distribution obtained via COMSOL Multiphysics simulation is presented, with the red boxed area highlighting the region of interest. To ensure accuracy, COMSOL simulation results were cross-validated with direct data export alongside image analysis outcomes. The red boxed area corresponds to the core electrolysis reaction zone, with boundary conditions defined by electrode spacing. Spatial heterogeneity of the electric field strength distribution was further characterized using ImageJ’s “Plot Profile” tool, confirming that this intensity is sufficient for efficient cell lysis while minimizing side effects.


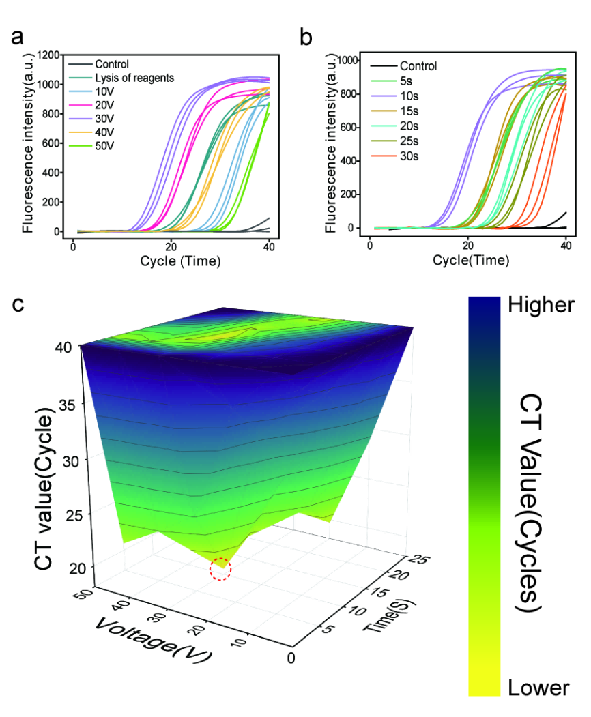


**Supplementary Fig. 6 | Optimization Experiments for Electro-Lysis.** (a) Comparison of qPCR results for electro-lysis under different voltage conditions (fixed duration of 10$s$) versus reagent-based lysis. The results clearly indicate that a voltage of 30$V$ yields the optimal outcome.(b) Comparison of qPCR results for electro-lysis under different durations (fixed voltage of 30$V$). The data shows that a duration of 10$s$ provides the best result.(c) Three-dimensional gradient plot summarizing data from (a) and (b). The plot compiles and organizes the results for various voltage conditions and corresponding durations. The peak point, marked by a red dashed circle, represents the optimal condition: a voltage of 30$V$ and a duration of 10$s$.


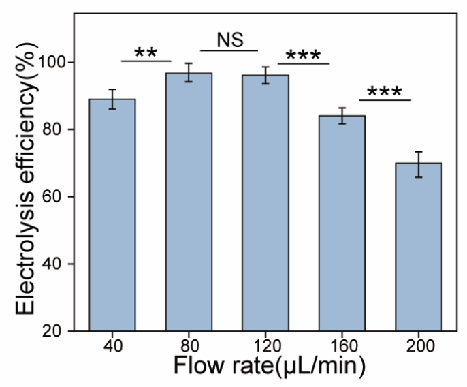


**Supplementary Fig. 7 |** Electro-lysis efficiency at different flow rates. Results depict the impact of flow velocity on cell lysis efficiency.


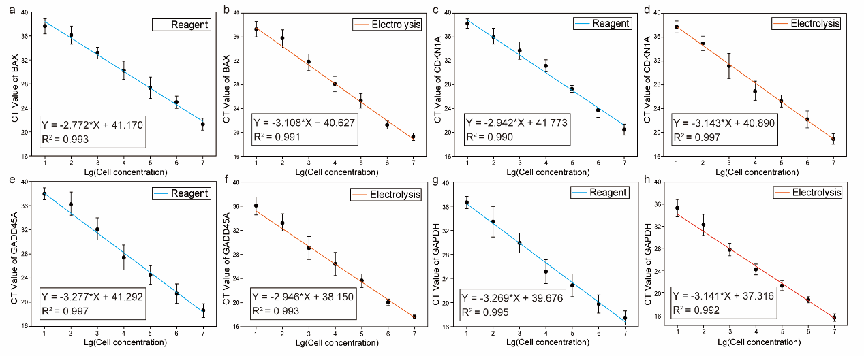


**Supplementary Fig. 8 | Concentration Linearity Fitting Equation.** Linear relationship between CT values and logarithmic cell concentration for reagent-based and electro-lysis methods. When processing cell samples of varying concentrations using reagent-based lysis and electro-lysis, the CT values of the four target markers—BAX, CDKN1A, GADD45A, and GAPDH—exhibit a linear relationship with the logarithm of cell concentration, as illustrated in the figure.


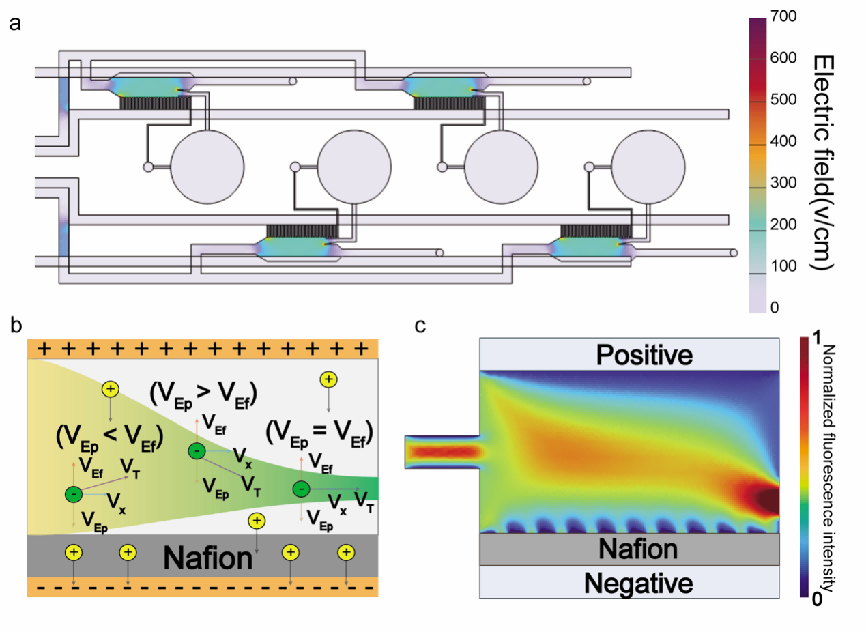


**Supplementary Fig. 9 | Principle and Simulation of Electro-Enrichment.** (a) Simulation of electric field strength in the electro-enrichment zone using COMSOL Multiphysics. The electric field strength across the four modules of the enrichment zone is approximately 150 V/cm. (b) Diagram illustrating the forces acting on various ions. Initially, all ions are subjected to an electric field force and a horizontal driving force. As cations accumulate on the inner side of the Nafion membrane, anions experience an attractive force from the cations.When anions are relatively close to the Nafion membrane, the osmotic force is less than the electric field force, causing anions to move toward the positive electrode.When anions are closer to the positive electrode, the osmotic force exceeds the electric field force, driving anions toward the Nafion membrane.At a specific position, these two forces balance each other, resulting in a net vertical force of zero. At this point, anions are only influenced by the horizontal driving force, achieving the enrichment effect.(c) Simulation of anion movement trends in the electro-enrichment zone using COMSOL Multiphysics.Initial conditions: voltage set at 30$V$, flow rate at 30$\mu L/min$, and the Nafion membrane assigned special boundary conditions (prohibiting passage of negatively charged ions).The steady-state simulation results, as shown in the figure, demonstrate that nucleic acid molecules are stably enriched in a fixed band, validating the feasibility of our hypothesis.

**
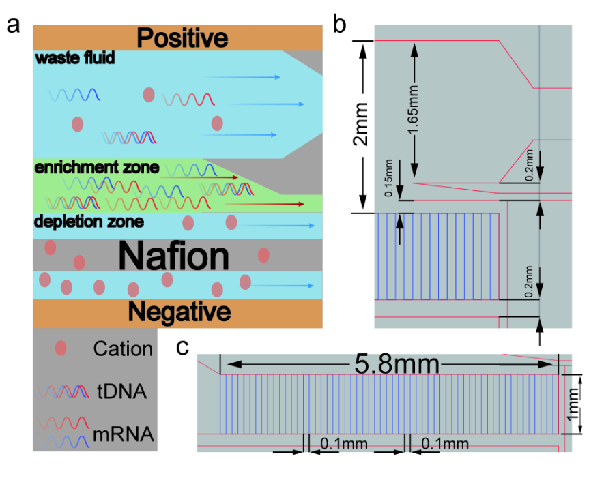
**

**Supplementary Fig. 10 | Design of "M"-Type Flow Channel and Nafion Slots.** (a) Principle of the "M"-type flow channel. Due to the presence of an electric field and the Nafion membrane (selective for cation permeation), ion enrichment occurs. The "depletion zone" is influenced by the applied electric field, where the electroosmotic force of cations repels anions, concentrating them in the enrichment region. The region where anions are concentrated, termed the "enrichment zone," facilitates the enrichment process, causing the majority of nucleic acids to flow along this band. At one end of this band, a collection solution is gathered and directed to the chip’s detection zone, while outlets are designed for the remaining portions to recover waste liquid. (b) Dimensions of the "M"-type flow channel. The overall width of the electro-enrichment chamber is 2 mm. The waste liquid channel has a width of 1.65 mm, the enrichment channel is 0.2 mm, the channel exiting the depletion zone is 0.15 mm, and the bottom channel collecting cations is 0.2 mm. Based on these dimensions, the concentration ratio of the enrichment chamber is calculated to be approximately 10-fold. (c) Dimensions of the array slots for Nafion modification.nAs shown, each slot is a small rectangle with a length of 1 mm and a width of 0.1 mm. The slots are spaced 0.1 mm apart, with a total of 29 slots available for Nafion material modification.


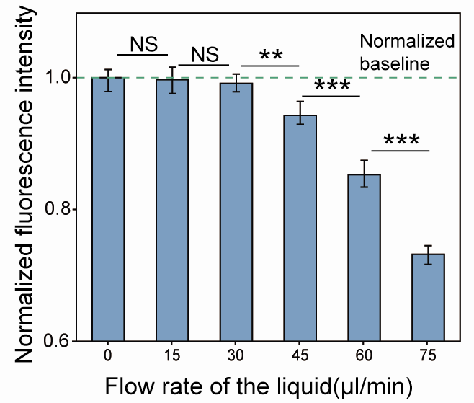


**Supplementary Fig. 11 |** Effect of flow rate on enrichment outcomes. Experiments reveal optimal results at flow rates below 30$\mu L/min$.


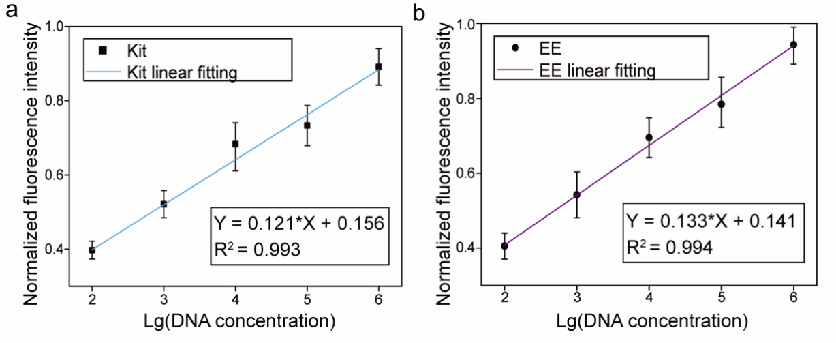


**Supplementary Fig. 12 | Relationship between Different DNA Concentrations and Enrichment Intensity.** (a) Linear relationship between reagent enrichment results and the logarithm of DNA concentration. (b) Linear relationship between electro-enrichment results and the logarithm of DNA concentration.

**
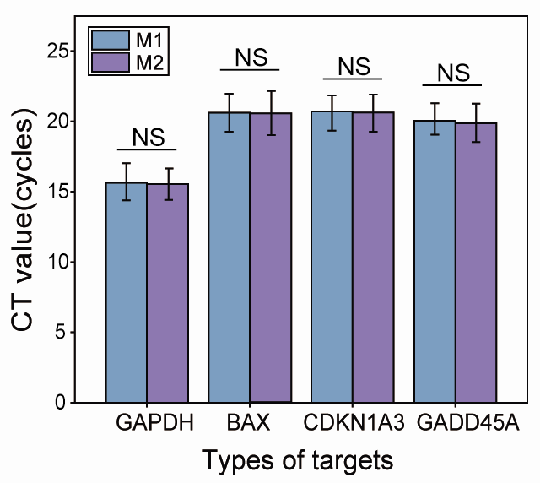
**

**Supplementary Fig. 13 |** Comparison of CT results between Module 1 (upper channel zone) and Module 2 (lower channel zone). Analysis of the CT values from both modules revealed no significant differences between the two.

**
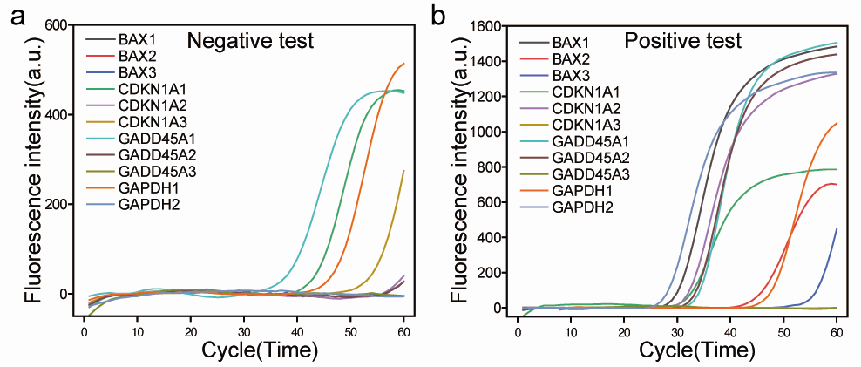
**

**Supplementary Fig. 14 | Primer Testing (Numbers at the end of primer names are solely for labeling and are random, with no additional significance).**(a) Negative testing.To achieve optimal detection performance in the experiment, multiple primers were designed and tested to screen for the most suitable ones for subsequent experiments.In the negative testing phase, each primer was evaluated using a standard LAMP reaction system. The samples used during testing consisted of two other targets excluding the primer's specific target (e.g., for testing BAX primers, the targets added were CDKN1A and GADD45A).Based on the negative testing results, the primers CDKN1A1, CDKN1A3, GADD45A1, and GAPDH1 were excluded due to unsatisfactory performance.(b) Positive testing.Each primer was further tested in the positive testing phase, where the standard LAMP reaction system was prepared and the corresponding target was added to its respective primer system (e.g., for testing BAX primers, the target added was BAX).Combining the results from the positive testing with those from (a), we ultimately selected the primer sequences BAX1, CDKN1A2, GADD45A2, and GAPDH2 for use in the experiments (sequence details are provided in Tables S1, S2, S3, and S4).


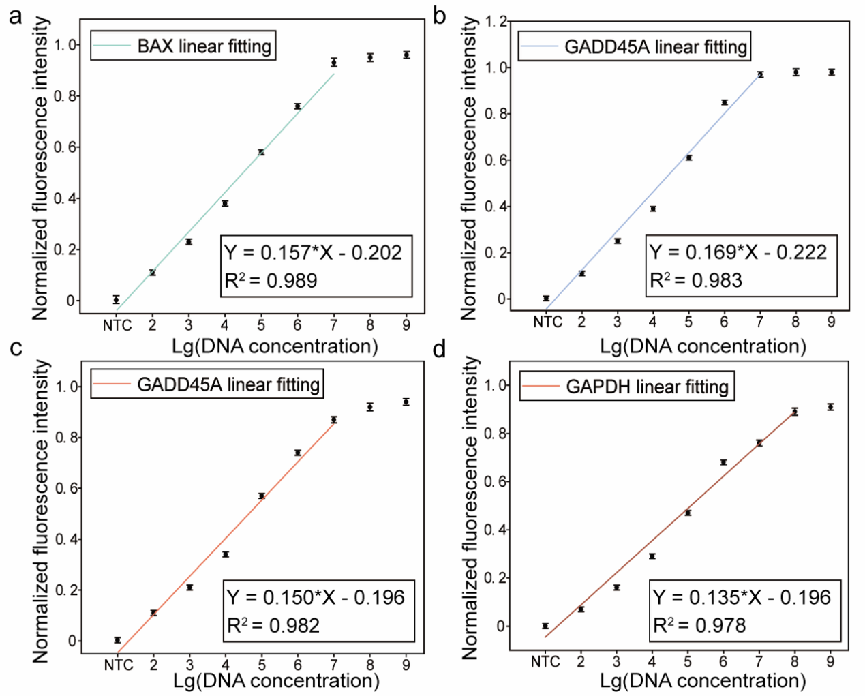


**Supplementary Fig. 15 | Fluorescence intensity within the chip-based detection system was quantitatively assessed.** (a) BAX standard plasmids (10^2^–10^9^ Copies/mL) were subjected to fluorescence detection, with intensities analyzed using ImageJ. A linear correlation analysis was performed between the fluorescence intensity and the logarithm of plasmid concentration, demonstrating a linear relationship within a certain range (with NTC considered as (X=1)). (b) CDKN1A standard plasmids (10^2^–10^9^ Copies/mL) were subjected to fluorescence detection, with intensities analyzed using ImageJ. A linear correlation analysis was performed between the fluorescence intensity and the logarithm of plasmid concentration, demonstrating a linear relationship within a certain range (with NTC considered as (X=1)). (c) GADD45A standard plasmids (10^2^–10^9^ Copies/mL) were subjected to fluorescence detection, with intensities analyzed using ImageJ. A linear correlation analysis was performed between the fluorescence intensity and the logarithm of plasmid concentration, demonstrating a linear relationship within a certain range (with NTC considered as (X=1)). (d) GAPDH standard plasmids (10^2^–10^9^ Copies/mL) were subjected to fluorescence detection, with intensities analyzed using ImageJ. A linear correlation analysis was performed between the fluorescence intensity and the logarithm of plasmid concentration, demonstrating a linear relationship within a certain range (with NTC considered as (X=1)).


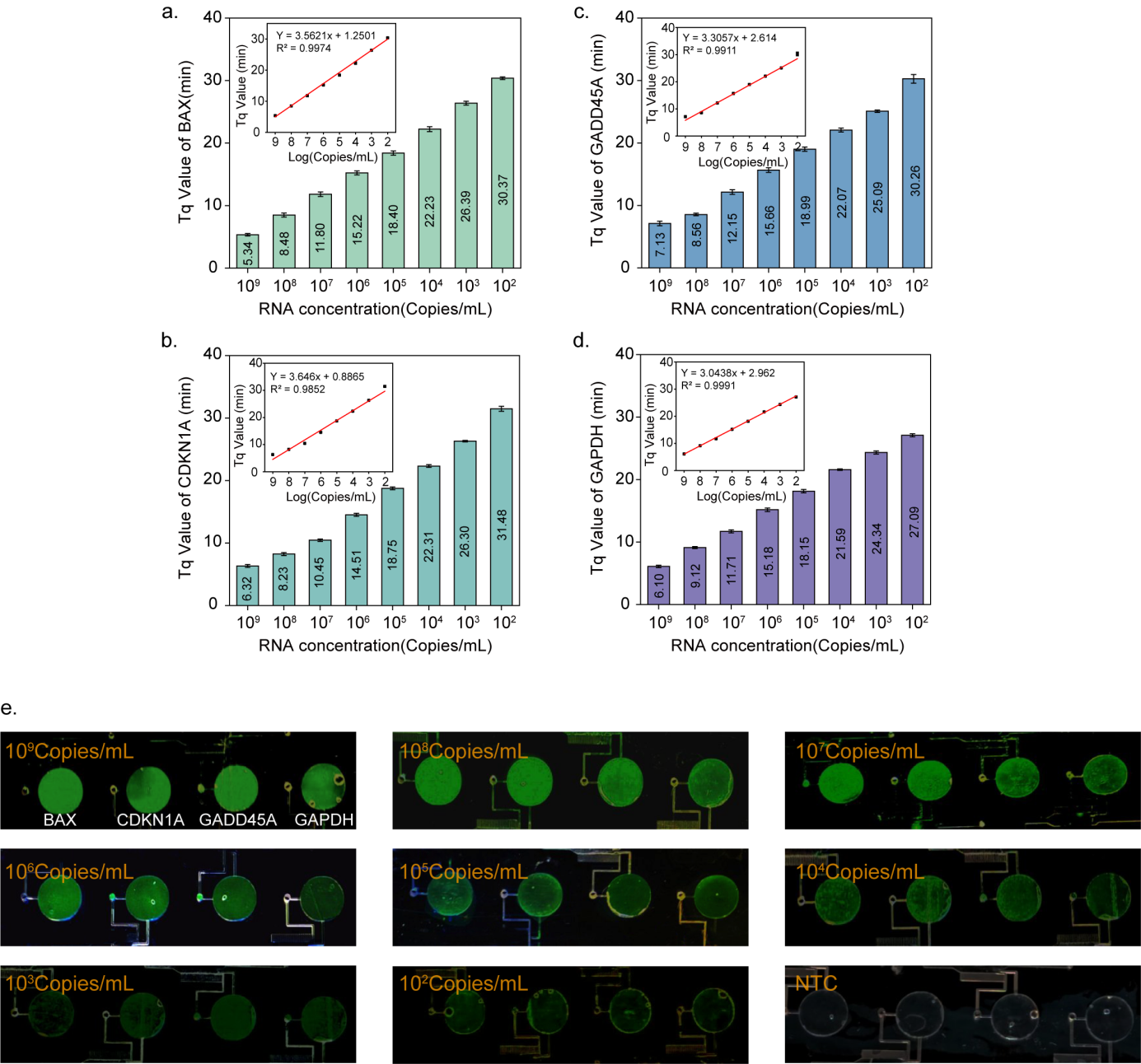


**Supplementary Fig. 16 | The detection performance of the EEMC by RNA plasmids.** (a-d) Sensitivity testing of LAMP targeting RNA plasmids of BAX (a), CDKN1A (b), GADD45A (c), and GAPDH (d). (e) Representative fluorescent images of the EEMC towards different concentrations of RNA plasmids from 10^2^ to 10^9^ copies/mL. NTC is the control group, with ddH₂O added to the system instead of plasmid.

**Table S1. ELISA Results for Reagent Lysis and Chip Lysis**

| well | 260 | 230 | 280 | $ng/\mu L$ | Ratio260/230 | Ratio260/280 |
| --- | --- | --- | --- | --- | --- | --- |
| Control1 | 0.001 | 0.001 | -0.0004 | 0.8 | 10 | -2.5 |
| Control2 | 0 | -0.0002 | 0.0001 | 0 | 0 | 0 |
| Control3 | -0.0005 | -0.0008 | -0.001 | -0.4 | 0.62 | 0.5 |
| Kit1 | 0.1479 | 0.0691 | 0.0684 | 118 | 2.04 | 2.16 |
| Kit2 | 0.1303 | 0.0606 | 0.0611 | 104 | 2.05 | 2.13 |
| Kit3 | 0.1417 | 0.0698 | 0.6561 | 113 | 2.03 | 2.16 |
| Chip1 | 0.1992 | 0.0976 | 0.0953 | 159 | 2.04 | 2.09 |
| Chip2 | 0.1791 | 0.0904 | 0.0856 | 143 | 1.98 | 2.09 |
| Chip3 | 0.1855 | 0.0918 | 0.0888 | 148 | 2.02 | 2.09 |

**Table S2. BAX PCR and LAMP Primers**

| Amplification | Primer | Sequence（5’-3’） |
| --- | --- | --- |
| PCR primers | Forward | TGAGCAGATCATGAAGACAGG |
|  | Reverse | TCCAGCCCATGATGGTTCT |
| LAMP primers | Fip | GCCACTCGGAAAAAGACCTCTCG-TGCTTCAGGGGATGATTGC |
|  | Bip | CGGGTTGTCGCCCTTTTCTACT-GATCAGTTCCGGCACCTTG |
|  | F3 | TGAGCAGATCATGAAGACAGG |
|  | B3 | TCCAGCCCATGATGGTTCT |
|  | F1c | GCCACTCGGAAAAAGACCTCTCG |
|  | B1c | CGGGTTGTCGCCCTTTTCTACT |
|  | F2 | TGCTTCAGGGGATGATTGC |
|  | B2 | GATCAGTTCCGGCACCTTG |
|  | Loop F | AGTCTGTGTCCACGGCG |
|  | Loop B | CCAGCAAACTGGTGCTCAA |

**Table S3. CDKN1A PCR and LAMP Primers**

| Amplification | Primer | Sequence（5’-3’） |
| --- | --- | --- |
| PCR primers | Forward | TCCTAAGAGTGCTGGGCATT |
|  | Reverse | GTCTGAGTGTCCAGGAAAGG |
| LAMP primers  LAMP primers | Fip | TAGCTGGCATGAAGCCGGC-TTAAAGCCTCCTCATCCCGT |
|  | Bip | CACTTGTCCGCTGGGTGGT-GAATTTCATAACCGCCTGTGAC |
|  | F3 | TCCTAAGAGTGCTGGGCATT |
|  | B3 | GTCTGAGTGTCCAGGAAAGG |
|  | F1c | TAGCTGGCATGAAGCCGGC |
|  | B1c | CACTTGTCCGCTGGGTGGT |
|  | F2 | TTAAAGCCTCCTCATCCCGT |
|  | B2 | GAATTTCATAACCGCCTGTGAC |
|  | Loop F | GGGAGAGAGGAAAAGGAGAAC |
|  | Loop B | TGTGGCTCCTTCCCATCG |

**Table S4. GADD45A PCR and LAMP Primers**

| Amplification | Primer | Sequence（5’-3’） |
| --- | --- | --- |
| PCR primers | Forward | TTTTTGCCGGGAAAGTCG |
|  | Reverse | TGTAGTTGAACTCACTCAGC |
| LAMP primers | Fip | CTTCAGTGCAATTTGGTTCAGTTAT-ACATGGATCAATGGGTTCC |
|  | Bip | ACCTTTGTAGTTACTCAAGCAGTT-CCCTTGGCATCAGTTTCT |
|  | F3 | TTTTTGCCGGGAAAGTCG |
|  | B3 | TGTAGTTGAACTCACTCAGC |
|  | F1c | CTTCAGTGCAATTTGGTTCAGTTAT |
|  | B1c | ACCTTTGTAGTTACTCAAGCAGTT |
|  | F2 | ACATGGATCAATGGGTTCC |
|  | B2 | CCCTTGGCATCAGTTTCT |
|  | Loop F | ACCGTTCAGGGAGATTAATCACT |
|  | Loop B | ACTCCCTACACTGATGCAAGGAT |

**Table S5. GAPDH PCR and LAMP Primers**

| Amplification | Primer | Sequence（5’-3’） |
| --- | --- | --- |
| PCR primers | Forward | GCAGCCGTTAGGAAAGC |
|  | Reverse | CGCCCAATACGACCAA |
| LAMP primers | Fip | AACTGGGCACGCACCGAGCTC-CCATCGGGCCAATCTCA |
|  | Bip | ACCAGGCGGCTGCGGAAAGCT-ACGTGCGCCCGTAA |
|  | F3 | GAACCAGCACCGATCACC |
|  | B3 | GCCCAAGGTCTTGAGGC |
|  | F1c | TTTTGGCTCCCCGCTGCAAG |
|  | B1c | GGGTCATCATCTCTGCCCCCT |
|  | F2 | ATACGTCGTAGAGTCCACCA |
|  | B2 | ATACTTCTCATGGCTCACGC |
|  | Loop F | ACGTAGGGGGGAAGGGA |
|  | Loop B | AAAAAAAGCGGGGAGAAAGTAGG |

**Movie S1.** Demonstration of the Enrichment Process in the Electro-Enrichment Zone.The video showcases the enrichment process in the electro-enrichment zone. The green fluorescence in the video represents a solution of FAM-labeled DNA molecules. According to the video’s orientation, the top side corresponds to the positive electrode, while the bottom side is the enrichment electrode (not visible within the video frame). The band below, completely separated from the green fluorescence, is the region modified with a Nafion membrane. The experimental conditions in the video are set at an applied voltage of 30$V$ and a flow rate of 30$\mu l/min$. The scale bar is indicated in the upper left corner of the video. The video begins with the power on, and the power state is toggled three times throughout the video to demonstrate the repeatability and stability of the experiment.
